# Influence of sedimentary deposition on the microbial assembly process in Arctic Holocene marine sediments

**DOI:** 10.3389/fmicb.2023.1231839

**Published:** 2023-08-28

**Authors:** Dukki Han, Tim Richter-Heitmann, Ji-Hoon Kim, Michael W. Friedrich, Xiuran Yin, Marcus Elvert, Jong-Sik Ryu, Kwangchul Jang, Seung-Il Nam

**Affiliations:** ^1^Department of Marine Bioscience, Gangneung-Wonju National University, Gangneung-si, Gangwon-do, Republic of Korea; ^2^Microbial Ecophysiology Group, Faculty of Biology/Chemistry, University of Bremen, Bremen, Germany; ^3^Marine Geology & Energy Division, Korea Institute of Geoscience and Mineral Resources, Daejeon, Republic of Korea; ^4^MARUM - Center for Marine Environmental Sciences, University of Bremen, Bremen, Germany; ^5^State Key Laboratory of Marine Resource Utilization in South China Sea, Hainan University, Haikou, China; ^6^Organic Geochemistry Group, Faculty of Geosciences, University of Bremen, Bremen, Germany; ^7^Department of Earth and Environmental Sciences, Pukyong National University, Busan, Republic of Korea; ^8^Division of Glacial Environment Research, Korea Polar Research Institute, Incheon, Republic of Korea

**Keywords:** sedimentary deposition, microbial assembly, Arctic Holocene, marine sediments, eDNA, metabarcoding

## Abstract

The sea-level rise during the Holocene (11–0 ky BP) and its resulting sedimentation and biogeochemical processes may control microbial life in Arctic sediments. To gain further insight into this interaction, we investigated a sediment core (up to 10.7 m below the seafloor) from the Chuckchi Shelf of the western Arctic Ocean using metabarcoding-based sequencing and qPCR to characterize archaeal and bacterial 16S rRNA gene composition and abundance, respectively. We found that Arctic Holocene sediments harbor local microbial communities, reflecting geochemical and paleoclimate separations. The composition of bacterial communities was more diverse than that of archaeal communities, and specifically distinct at the boundary layer of the sulfate–methane transition zone. Enriched cyanobacterial sequences in the Arctic middle Holocene (8–7 ky BP) methanogenic sediments remarkably suggest past cyanobacterial blooms. Bacterial communities were phylogenetically influenced by interactions between dispersal limitation and environmental selection governing community assembly under past oceanographic changes. The relative influence of stochastic and deterministic processes on the bacterial assemblage was primarily determined by dispersal limitation. We have summarized our findings in a conceptual model that revealed how changes in paleoclimate phases cause shifts in ecological succession and the assembly process. In this ecological model, dispersal limitation is an important driving force for progressive succession for bacterial community assembly processes on a geological timescale in the western Arctic Ocean. This enabled a better understanding of the ecological processes that drive the assembly of communities in Holocene sedimentary habitats affected by sea-level rise, such as in the shallow western Arctic shelves.

## Introduction

Microbial biogeography and the principles driving microbial assembly have been investigated in various environments (Martiny et al., 2006). Microbial biogeography is generally caused by four fundamental processes (Hanson et al., 2012): environmental selection (adaptation to specific conditions), ecological drift (random population fluctuations), dispersal ability (movement between locations), and speciation (formation of distinct species). Details of terminologies were described in Supplementary Information (ecological terminology). A recent change in perspective has been debated to differentiate contemporary microbial biogeography from that of the past environment; particularly, the dispersal of microbial populations was suggested as a critical criterion for such a differentiation (Martiny et al., 2006; Hanson et al., 2012). Indeed, endospores of thermophilic bacteria in marine sediments were suggested as a possible tracer to discern the dispersal of microbial populations from the past environment (Müller et al., 2014; Hanson et al., 2019). Dispersal ability with dispersal limitation (or priority effect) (Hanson et al., 2012) may be useful in describing microbial biogeography in marine sediments. For example, the movement of microbial populations or colonization of habitats by microbial populations can be mediated through passive or active mechanisms during sediment deposition. Prior occupation of early deposited layers by particular microbial populations either precludes new colonization by other microbes or results in competition between formerly established microbes and later successional microbes (the so-called priority effect). This assumption was recently tested using endospore-forming bacteria in Arctic fjord surface sediments (Hanson et al., 2019).

If microbial populations established in response to sedimentary conditions in surface sediments have survived or been conserved after sedimentation-induced burial (Starnawski et al., 2017), analyzing layer-specific microbial communities can provide information about their habitat conditions induced by past oceanographic changes. Recent studies have shown that microorganisms in subsurface sediments, whether alive or dead, can be used to decipher the paleo-depositional conditions that were prevalent when those microorganisms were buried (Han et al., 2017; Orsi et al., 2017; Vuillemin et al., 2018; Hanson et al., 2019; More et al., 2019, 2021). Orsi (2018) have shown that past oceanographic as well as depositional conditions seem to be represented by microbial communities, at least in non-advective marine sediments. However, these studies have primarily considered the post-burial existence of some subsisting taxa that employ survival strategies (e.g., switching to dormancy or less-competitive substrates) rather than investigating the community-wide changes in subsurface sediments influenced by changing depositional conditions. In the community level, quantification of the relative influences of stochastic (ecological drift or dispersal ability) and deterministic (environmental selection) processes has become a common method in microbial ecology (Stegen et al., 2012; Lindemann et al., 2013; Wang et al., 2013; Dini-Andreote et al., 2015; Stegen et al., 2015; Feng et al., 2018; Tripathi et al., 2018; Han et al., 2020; Richter-Heitmann et al., 2020; Han et al., 2021). This quantification has established a comprehensive framework to ecologically explain the assembly of microbial communities (see the ecological terminology in Supplementary Information).

Microbial communities of the Arctic Ocean have been studied extensively in the Chukchi Sea (western Arctic Ocean) (Boeuf et al., 2013; Han et al., 2014, 2015, 2016, 2017; Dong et al., 2017). In the contemporary environment of the Chukchi Sea, the trends of biogeographic patterns of microbial habitats from seawaters to surface sediments are associated with sea-ice dynamics (Han et al., 2014, 2015, 2016). In general, microbial ecology in marine sediments is controlled by sedimentation rate, organic matter content, and oxygen level. In contrast to the contemporary environment, the Chukchi Sea was glaciated due to the lower sea level of *ca.* 120 m at the Last Glacial Maximum (LGM; *ca.* 21.5 ky BP) and geologically conserved because of lesser physiochemical disturbances before the opening of the Bering Strait (Jakobsson et al., 2014). The influence of the opening of the Bering Strait on the sedimentation, organic matter content, and the oxygenic euphotic zone dramatically changed in response to the Holocene (11–0 ky BP) sea-level rise.

In the western Arctic Ocean, oceanographic structure drives the primary production and microbial habitat in the euphotic zone (Monier et al., 2015) and its subsequent deposition may directly or indirectly influence the assembly of microbial communities in the seafloor. The nutrient-rich and low salinity waters from the North Pacific Ocean flow into the western Arctic Ocean *via* the Bering Strait (Cooper et al., 1997). The inflow of North Pacific water (or Bering Strait Inflow) plays an important role in controlling the influx of heat, freshwater, and nutrients into the western Arctic water column (Woodgate and Peralta‐Ferriz, 2021). This process is critical for the rapid reduction in sea-ice content and changes in the western Arctic Ocean circulation caused by recent global warming (Ortiz et al., 2009; Stein et al., 2012); thus, the hydrography of the western Arctic Ocean has been strongly influenced by the intensity of the Bering Strait Inflow (BSI). Tracking the BSI signal aids the understanding of the variations in sea-ice content and marine production in the western Arctic Ocean (Stein, 2008; Yamamoto et al., 2017; Han et al., 2018). The fate of the BSI is linked to sea-level fluctuations at geological timescales and consequently to paleo-primary production, for which the inflow from the nutrient-rich Pacific Ocean. The BSI may have controlled biogeochemical processes and subsequently the microbial life in the Arctic Ocean on a geological timescale (Han et al., 2017).

The sedimentation rate in the Chukchi Sea varied with sea-level rise during the Holocene (Stein et al., 2017). Microbial biogeography in Arctic Holocene sediments (Han et al., 2017) suggests that past oceanographic changes can be inferred from microbial habitat tracking with traditional paleoclimate records. Nonetheless, the interplay between dispersal limitation and environmental selection, which together or alone shape microbial biogeography in response to the sea-level rise and its resulting sedimentary deposition, has not been investigated for Arctic subsurface sediments. In this study, we estimate the influences of fundamental assembly principles on microbial communities in Holocene sediments beneath the permanently stratified and non-advective seafloor of the Chukchi Sea. The primary goals of the present study are to identify microbial habitats in Holocene Arctic subsurface sediments and to understand their assembly processes and ecological succession on a geological timescale.

## Materials and methods

### Core description and geochemical properties

A sediment core ARA06C-JPC01(i.e., JPC1; 73° 37.2217′ N / 166° 25.7329′ W; water depth: 100 m) was retrieved from the Chukchi Sea inner shelf during the expedition of the Ice Breaking Research Vessel (IBRV) *ARAON* in 2015. JPC1 was obtained after coring up to 10.7 m below the seafloor (mbsf). Sediment samples were immediately sealed on board and stored under refrigeration before further geochemical and microbial analyses. JPC1 exhibits three distinct lithological units, which comprise bioturbated mud with dark mottles (0–7.6 m), laminated mud (7.6–9.1 m) and massive mud (9.1–10.3 m), and detailed description of JPC1 was explained in Supplementary Information (core description). Paleo-oceanographic changes at the coring site of JPC1 were previously described using sedimentary records (< 10 ky BP) that indicate a sea-level rise in the western Arctic Ocean during the Holocene (Stein et al., 2017).

The age model of JPC1 was determined by accelerator mass spectrometry (AMS) ^14^C dating conducted at the site of ARA2B-1A, which is 3.2 km away from the site of JPC1 (Stein et al., 2017). This model is based on the comparison between JPC1 and ARA2B-1A profiles of wet bulk density (WBD) and magnetic susceptibility (MS) (Supplementary Figure S1). In geochemical tests, major and trace elements and total organic carbon (TOC) in the solid phase were determined using inductively couple plasma optical emission spectroscopy (ICP-OES) and Rock-Eval analyses at 30 cm depth intervals (*n* = 35, each 1 g dried sediment) for JPC1. Headspace methane and pore water chemistry (liquid phase) were selectively presented by Kim et al. (2021). Details of geochemical experiments were described in Supplementary Information (geochemical experiment).

### DNA extraction, metabarcoding, and qPCR

A total of 177 sediment samples were collected from JPC1 at 5–10 cm depth intervals, all of which were freeze-dried. Environmental DNAs (eDNAs) were extracted from 0.25 g of dry weight sediment using the MOBIO’s PowerSoil DNA extraction kit and further purified using PowerClean Pro DNA Clean-Up Kit (MOBIO, Carlsbad, CA, United States) according to the manufacturer’s instructions. The concentration of eDNAs was measured using the Quant-iT PicoGreen dsDNA Reagent (Molecular Probes, Eugene, OR, United States).

The extracted eDNAs were prepared for metabarcoding-based sequencing with a two-step PCR (amplicon and index PCR) as previously described (Han et al., 2020). Briefly, eDNAs were PCR-amplified in triplicates using primers specific to archaeal (Baker et al., 2003) and bacterial (Herlemann et al., 2011) 16S rRNA genes. The amplified 16S rRNA gene fragments were used for the index PCR and pooled prior to further sequencing using the Illumina MiSeq platform (Macrogen, Seoul, South Korea). Metabarcoding sequence data were submitted to the NCBI Sequence Read Archive^1^ under the accession number PRJNA614491. Archaeal and bacterial abundance was determined using real-time quantitative PCR (qPCR) with general archaea- and bacteria-and methanogen-specific primers. Details of PCR conditions and sequences of primers for metabarcoding and qPCR are provided in the Supplementary Information (metabarcoding and qPCR).

## Sequence processing, phylogenetic analyses, and statistics

The sequences were analyzed using the Mothur software package (v.1.40.5) (Schloss et al., 2009) according to the Mothur MiSeq pipeline (Kozich et al., 2013) to profile the composition of microbial assemblages and to elucidate the diversity (alpha and beta) of archaeal and bacterial assemblages. Sequence quality filtering was first carried out with singleton removal and correction of amplification and sequencing errors. The filtered sequences were normalized (randomly subsampled) to 10,000 sequences per sample, and then taxonomically identified against the SILVA SSU database (v132) before clustering of sequences into the operational taxonomic units (OTUs).

OTUs were determined from the archaeal and bacterial sequences at 97% similarity level according to the Mothur pipeline (Kozich et al., 2013). Bacterial (*n* = 1,730,000) and archaeal (*n* = 495,000) sequences were clustered into their respective OTUs. Two asymptotic species richness estimators (Chao and Chiu, 2016) such as Chao1 and abundance-based coverage estimator (ACE) were calculated with OTUs using the ‘summary.single’ command in Mothur to estimate the alpha diversity (species richness). The beta diversity of archaeal and bacterial OTUs was calculated using Mothur (theta Yue and Clayton (YC) distance) and R (Bray-Curtis distance) program version 3.5.3,^2^ respectively. Briefly, the beta diversity was visualized in non-metric multidimensional scaling (NMDS) (i) with total OTUs using the nmds command in Mothur (Kozich et al., 2013) or (ii) with relative abundance of selected OTUs (the percent composition of sequences for the most abundant 300 bacterial or 459 archaeal OTUs against the total number of sequences in each samples; described below) using the metaMDS function of the R vegan package (Oksanen et al., 2022). A significant difference in the separated clusters in NMDS was evaluated by analysis of molecular variance (AMOVA) using the amova command in Mothur (Kozich et al., 2013) or by multiple response permutation procedure (MRPP) using the mrpp function of the vegan package. Permutational multivariate analysis of variance (PERMANOVA) was performed using the adonis function with the Bray-Curtis method, and its post-hoc pairwise comparison was performed using the pairwise.adonis function with the false discovery rate method in the vegan package. Correlation analysis between the relative abundance of methanogenic archaeal sequences and copy number of mcrA gene was applied using the cor function (spearman method) of the R stats package (R Core Team and C Worldwide, 2002).

From the OTU clustering, 16,386 bacterial and 459 archaeal OTUs were deduced. Owing to limited computing resources for the index calculation with Phylocom (Webb et al., 2008), the bacterial dataset was reduced to 300 most abundant OTUs (82.9% of total bacterial sequences). 300 bacterial and 459 archaeal OTUs were employed to determine the extent of influence of habitat specificity or biological interaction on the assembly processes using net relatedness index (NRI) and nearest taxon index (NTI). These OTUs were employed to predict the relative influence of deterministic and stochastic processes in bacterial communities using βNTI. Briefly, the Phylocom program was used using 300 bacterial and 459 archaeal OTUs to generate NRI, NTI, and βNTI for all sediments, and Bray–Curtis-based Raup–Crick metric (RC_bray_) was calculated (Stegen et al., 2013) using 300 bacterial OTUs for pairwise comparisons with |βNTI| < 2 using ‘picante’ package (Kembel et al., 2010) in R. Details of phylogenetic analysis are provided in Supplementary Information (phylogenetic analysis).

## Results

### Pore water and solid phase geochemistry related to paleoclimate phases

JPC1 was divided into sediment sections above 7.6 mbsf based on the correlated profiles of WBD and MS with reference sediment core (ARA2B-1A) (Supplementary Figure S1). The deeper section of JPC1, below 7.6 mbsf, exhibited over-consolidated sediments characterized by remarkably high WBD values (Supplementary Figure S1), indicative of diamicton resulting from grounded ice in the western Arctic Ocean (Stein et al., 2017). For the paleoclimate separation of JPC1 above 7.6 mbsf, a modified procedure as previously described was applied (Supplementary Figure S1) (Stein et al., 2017). The upper sediment section above 1.20 mbsf was likely deposited during the Late Holocene (LH; 3.7 ky BP ~ present). The Middle Holocene sediments were identified between 1.25 mbsf and 6.05 mbsf in JPC1 (MH; 8.0 ~ 3.7 ky BP), while the lower section, starting at 7.6 mbsf, is referred to as the Early Holocene sediments (EH; 11.0 ~ 8.0 ky BP). The over-consolidated section below 7.6 mbsf is referred to as Deglaciation (DG; ~ 11.0 ky BP) after the LGM (Last Glacial Maximum).

**Figure 1:**
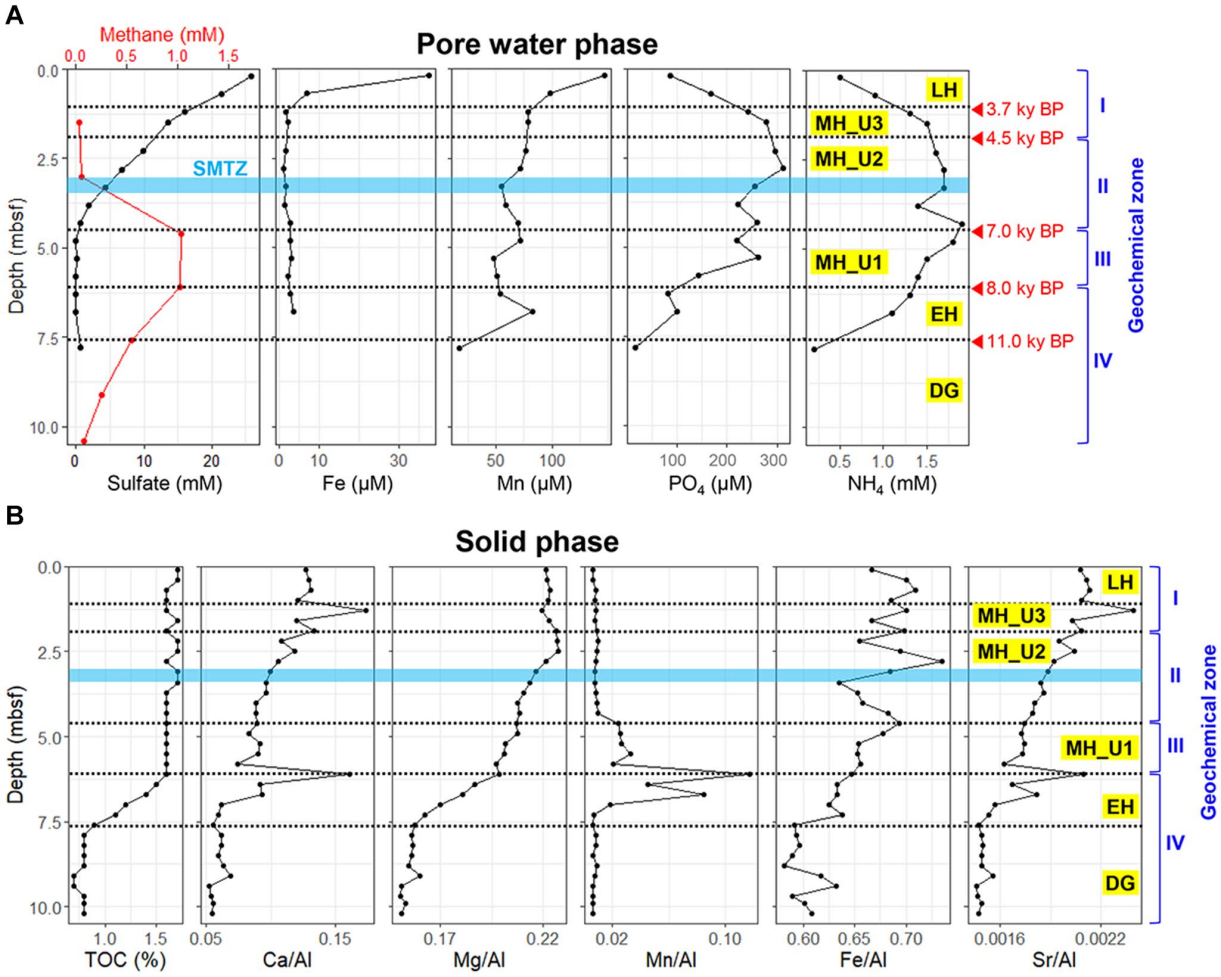
Geochemical profiles of JPC1. **(A)** Headspace methane and pore water profiles of sulfate, iron, manganese, phosphate, and ammonium. **(B)** Elemental composition profiles in sediment under paleoclimate phase. JPC1 was separated into Late Holocene (LH), Units of Mid Holocene (MH_U3, MH_U2, and MH_U1), Early Holocene (EH), and Deglaciation (DG) under the paleoclimate phases and geochemical zones.

The geochemical properties of JPC1 display clear distinctions corresponding to different paleoclimate phases (Figure 1). Particularly, within the MH section, the profiles of sulfate and methane concentration were further segregated into Unit3, Unit2, and Unit1 based on the presence of the sulfate–methane transition zone (SMTZ). The geochemical sections of JPC1 were as follows: LH: top–1.20 mbsf; geochemical zone I: sulfate reduction, MH Unit3 (MH_U3: 1.25–2.15 mbsf; geochemical zone I: sulfate reduction), MH Unit2 (MH_U2: 2.20–4.35 mbsf; geochemical zone II: sulfate–methane transition), MH Unit1 (MH_U1: 4.40–6.05 mbsf; geochemical zone III: methanogenesis), EH: 6.10–7.55 mbsf; geochemical zone IV: methanogenesis, and DG: 7.60 mbsf–bottom; geochemical zone IV: methanogenesis. In the uppermost sediments (> 20 cmbsf), the concentrations of dissolved iron (Fe: 37.5 μM) and manganese (Mn: 146.4 μM) were highest due to diagenetic reduction of their oxide minerals, while concentrations of sulfate, ammoium, and phosphate were 25.8 mM, 0.5 mM, and 87.1 μM, respectively, (Figure 1A). Below geological zone I, the concentrations of Fe (~1.6 μM) and Mn (~77.1 μM) sharply decreased in accordance with organoclastic sulfate (~13.6 mM) reduction dominating in the sediments. The concentrations of ammonium (1.6 ~ 1.9 mM) and phosphate (260.5 ~ 295.5 μM) in the pore water gradually increased along the core with a decrease in sulfate (~0.7 μM) concentration (geological zone II). In contrast to the sulfate profile, methane concentration increased with depth in the MH_U2 section (geological zone II) and reached its maximum (~1.0 mM) in the MH_U1 section below the SMTZ (geological zone III). The maximum methane concentration decreased by 0.08 mM in the lowermost sediments (geochemical zone IV: EH and DG).

**Figure 2:**
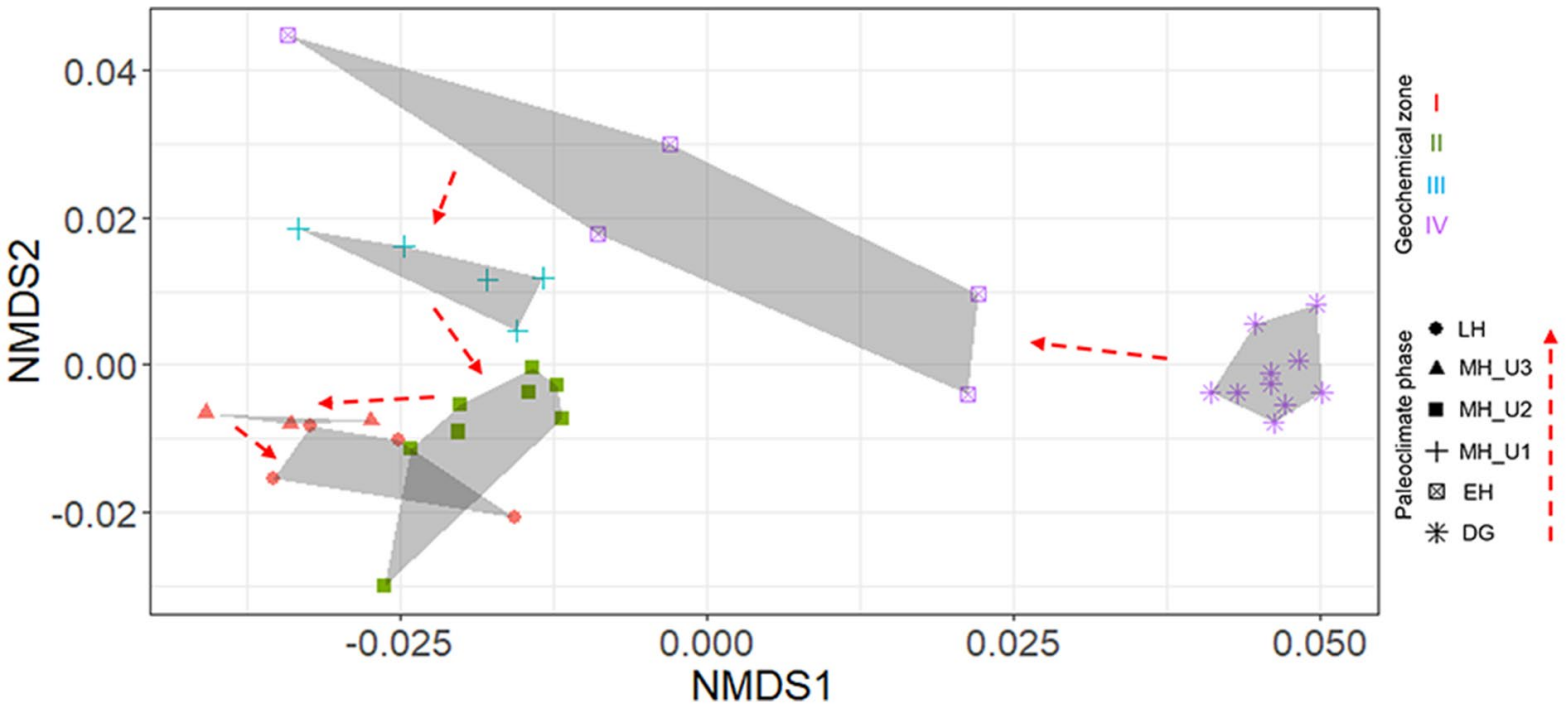
Element (*n* = 50) separation of sediments from JPC1 into paleoclimate phases using NMDS. A significant difference among paleoclimate separations was visualized using NMDS and determined by MRPP (*p* < 0.001). In NMDS, paleoclimate phases were identified as each symbol, and geochemical zones were also visualized with colors (red: I, dark green: II, light blue: III, light violet: IV).

Although pore water extraction from the lowermost section was failed due to over-consolidated sediments (DG section), solid-phase geochemistry showed that DG sediments were distinctly separated from the others (Figure 1B). In general, the solid phase biogeochemistry (element/Al and TOC) exhibited a clear separation of the DG section from the other sections, charactized by the lowest values of TOC and other elements. Such distinct patterns in geochemical properties, in both liquid (pore water) and solid (sediment) phases, strongly suggest that sedimentary conditions between the deglaciation and the Holocene can be distinguished under different paleoclimate phases in JPC1. To validate this assumption, we conducted a statistical analysis using the ICP-OES element dataset (50 elements, supplementary dataset) in NMDS with Bray-Curtis distance and MRPP. NMDS revealed distinguishable separations as inferred from the geochemical zones and paleoclimate phases (Figure 2), and a significant difference was found among the inferred separations using MRPP (*p* < 0.01) (Supplementary Table S1). In particular, the mean distance within each group in MRPP supported the clustering patterns observed in the NMDS ordination. For example, the relatively loose clusters of geochemical zone IV and paleoclimate phase EH in NMDS exhibited longer distances (IV: 0.23 and EH: 0.37) compared to the other groups (< 0.09). Interestingly, the clusters of paleoclimate phases in the NDMS ordination demonstrated a more analogous pattern to the geological timescale (as indicated by the red dashed arrows in NMDS).

### Microbial biogeography in Arctic Holocene sediments

**Figure 3:**
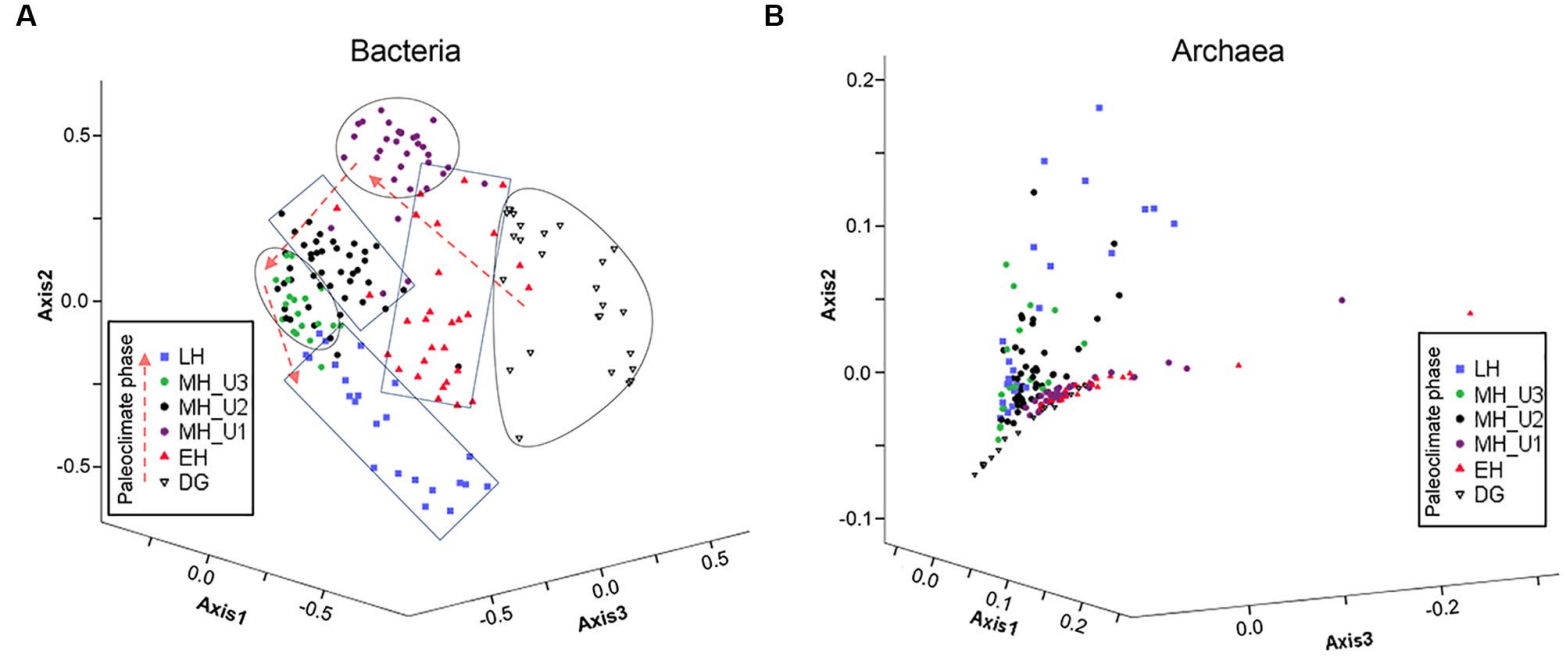
Expected microbial habitats of JPC1. **(A)** Bacterial and **(B)** Archaeal communities are displayed with beta diversity pattern in different paleoclimate phases using NMDS, and the significance of differences in bacterial and archaeal microbial communities between each pair of paleoclimate phases are estimated using AMOVA (Supplementary Table S2).

Based on the inferred sedimentary conditions from the ICP clusters (Figure 2), microbial biogeography in JPC1 sediments was surveyed using eDNA metabarcoding (*n* = 177). Nortably, the copy number of the archaeal 16S rRNA gene was much higher than that of the bacterial 16S rRNA gene. However, the distribution of eDNAs was similar to the bacterial 16S rRNA gene rather than to the archaeal 16S rRNA gene (Supplementary Figure S2). Analysis of beta diversity (Figure 3 and Supplementary Table S2) showed that both bacterial and archaeal communities were significantly separated in each paleoclimate phase (AMOVA, *p* < 0.001 in all comparison pairs), albeit this paleoclimate-specific clustering was less pronounced for archaea. Similar pattern were observed in this beta diversity among the geochemical zones (Supplementary Figure S3). Regarding alpha diversity, species richness (Chao1 and ACE) was analyzed using box plot analysis under paleoclimate phases and geochemical zones (Supplementary Figure S4). The indices of species richness in the box plot showed a depth-wise distribution, although the lower and upper quartiles of each paleoclimate phase and geochemical zone overlapped with each other. Given that speciation can lead to differences in species richness among habitats (Stegen et al., 2013), the depth-wise distribution of alpha diversity suggests a weak influence of speciation among paleoclimate phases and geochemical zones in JPC1.

**Figure 4:**
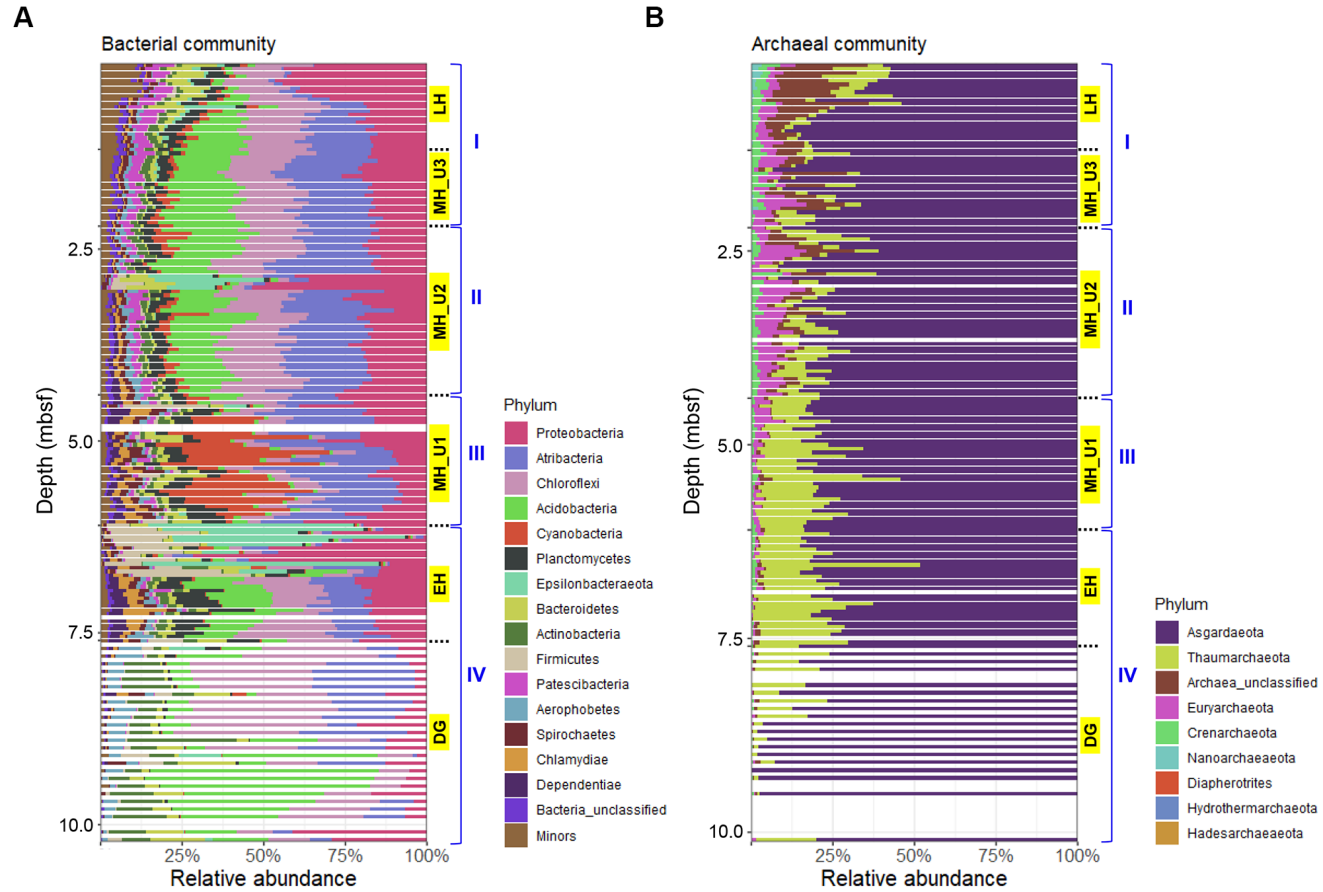
Composition of microbial assemblages in JPC1. **(A)** Bacterial and **(B)** Archaeal communities. Taxonomy of minor populations was described in supplementary dataset.

**Figure 5:**
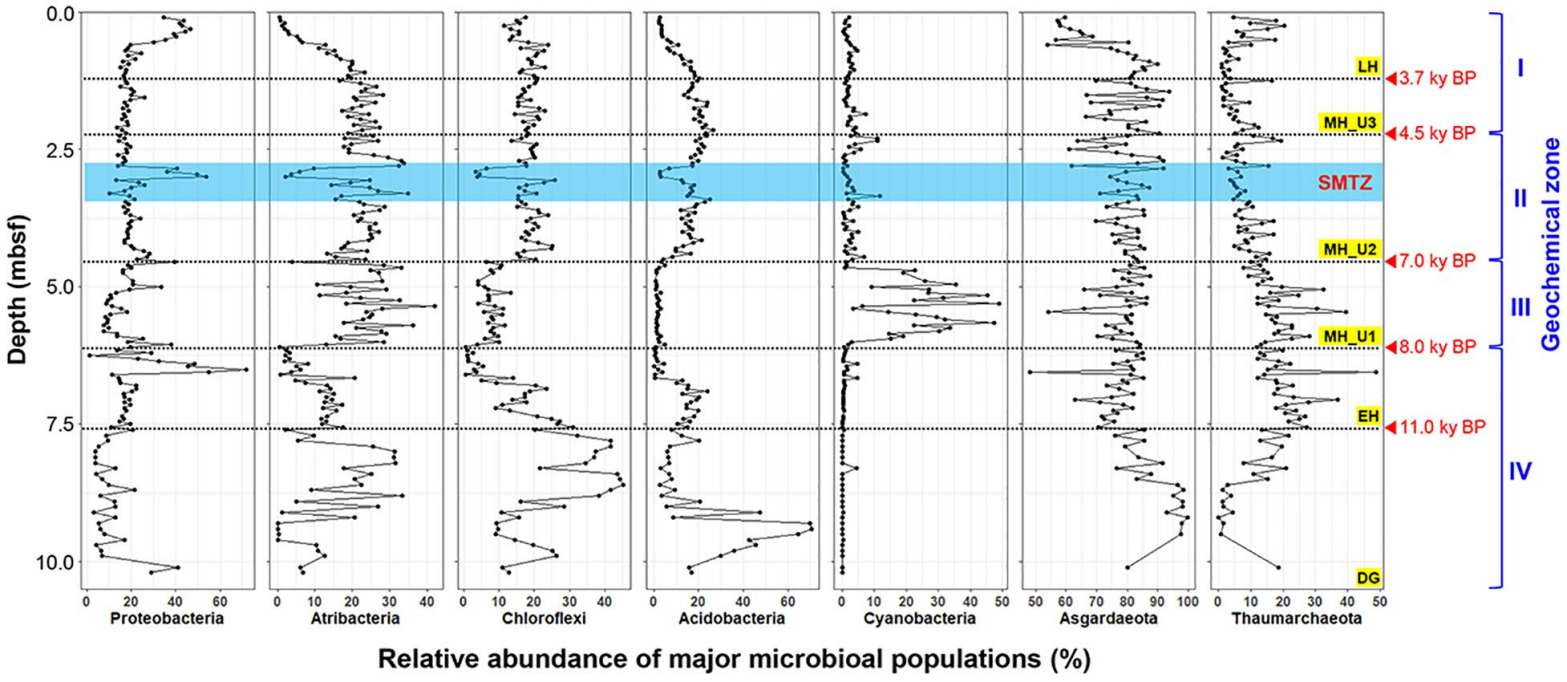
Relative abundance of major populations in microbial assemblages of JPC1.

The bacterial communities in JPC1 exhibited higher diversity compared to the archaeal communities (Figure 4). Proteobacteria (19.5%), Atribacteria (17.4%), Chloroflexi (16.4%), Acidobacteria (13.0%), and Cyanobacteria (5.0%) were the most abundant (Figure 4A), and their distribution showed distinct spatial patterns in JPC1 (Figure 5). For example, the distribution of Proteobacteria sharply decreased from the surface to the LH section (or geochemical zone I: sulfate reduction). Nortably, 35.4% of Proteobacteria sequences were identified as known sulfate-reducing bacteria, including Desulfobacterales (17.5%), Desulfarculales (14.7%), Desulfuromonadales (1.6%), Syntrophobacterales (1.4%), and Desulfovibrionales (0.1%). Atribacteria and Chloroflexi, which can produce H_2_ *via* fermentation and make it available to sulfate-reducing bacteria or methanogens (Orsi, 2018), showed similar distribution patterns in JPC1, and they are dominant members of the Arctic sedimentary subsurface (Jorgensen et al., 2012; Treude et al., 2014; Han et al., 2017). In contrast, the relative abundance of Acidobacteria, known for their ability to consume H_2_ (Greening et al., 2015; Giguere et al., 2021), was remarkably high in the lowermost sediments in JPC1. Such a high abundance is commonly observed in Arctic permafrost and tundra (Männistö et al., 2013; Steven et al., 2013). Cyanobacteria and oxygenic photosynthetic bacteria were also prominently featured in the MH_U1 section. This finding aligns with a recent report of cyanobacterial sequences in Holocene sediments (<5.2 ky BP) in the Black Sea (More et al., 2019), supporting the possibility of sedimentary ancient DNA of cyanobacteria in the Arctic Ocean (De Schepper et al., 2019). On the contrary, archaeal communities mostly consisted of Asgardaeota (79.0%) and Thaumarchaeota (11.8%) (Figure 4B), displaying an antiparallel distribution pattern in JPC1 (Figure 5). We found methanogenic archaeal sequences (4.53%) such as Methanomassiliicoccales (2.92%), unclassified Bathyarchaeia (1.41%), Methanocellales (0.09%), Methanosarcinales (0.07%), Methanomicrobiales (0.05%), and Methanofastidiosales (< 0.01%). These methanogenic archaeal sequences were partially enriched at geochemical zone II (SMTZ: 2.20–4.35 mbsf) (Supplementary Figure S5), and their downward distribution was significantly correlated with the abundance of *mcrA* gene (spearman correlation coefficient: 0.61, *p* < 0.01), a marker of methanogenesis. Overall, the relative abundance of major populations in microbial assemblages remarkably matched the geochemical zones in JPC1. However, none of the archaeal and bacterial taxa from the microbial community independently represent the successional variation of paleoclimate phases, as shown by the beta diversity pattern.

### Assembly process of microbial community related to paleoclimate phases

**Figure 6:**
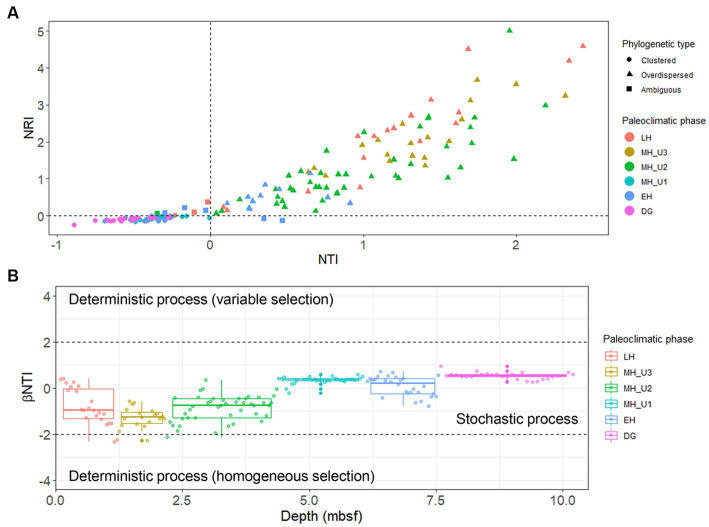
Ecological assembly perspectives estimated with selected 300 bacterial OTUs. **(A)** Separation into phylogenetically overdispersed (NRI and NTI < 0), clustered (NRI and NTI > 0), or ambiguous (NRI > 0 and NTI < 0 or NRI < 0 and NTI > 0) types using NTI and NRI indexes and their distribution in the paleoclimate phases (Supplementary Table S3). **(B)** Separation into stochastic and deterministic assembly processes using βNTI index in different paleoclimate phases. The beta diversity pattern of the selected 300 bacterial OTUs (Supplementary Figure S6) was directly comparable to that of the whole bacterial community (Figure 3).

The study used a total of 300 selected bacterial OTUs, with beta diversity patterns (Supplementary Figure S6) similar to that of the whole bacterial community (Figure 3), along with 459 archaeal OTUs to estimate the influence of the assembly process on microbial communities under various paleoclimate phases. For the bacterial OTUs, the majority of the total 172 JPC1 sediment-specific communities were assigned as either phylogenetically overdispersed (*n* = 90; NRI and NTI < 0), indicating the presence of biological interactions (such as symbiosis–competition or immigration–emigration) governing local assembly, or as clustered type (*n* = 74; NRI and NTI > 0), reflecting habitat specificity (environmental selection) in the NRI-NTI model (Webb et al., 2002; Amaral-Zettler et al., 2011; Han et al., 2020, 2021). A small of sediments (eight) remained ambiguous (NRI > 0 and NTI < 0 or NRI < 0 and NTI > 0). Interestingly, the variation in phylogenetic patterns was distinct among different paleoclimate phases (Figure 6A and Supplementary Table S3). For example, the phylogenetic over-dispersion type was predominant with an increased depth with 74% of the bacterial OTUs assembled into LH, 100% in MH_U3, and 98% in MH_U2, demonstrating the influence of biological interactions with microbial processes like sulfate reduction, methanogenesis, and fermentation. Thereafter, the MH_U1 section was overwhelmingly associated with the phylogenetically clustered type (97%), as was the DG section (100%). However, the in-between (EH) clustering (46%) and over-dispersion (36%) were relatively balanced, except for the ambiguous cases (18%).

This variation under different paleoclimate phases was further corroborated through subsequent βNTI modeling. The values of βNTI in JPC1 communities almost entirely ranged between 2 and − 2, indicating a dominat stochastic assembly of bacterial communities in Arctic Holocene sediments (Figure 6B). Considering the patterns observed in the NRI-NTI model, however, the variation of βNTI values from the bottom (DG) to top (LH) sections suggests that bacterial communities in JPC1 follow different assembly patterns under distinct paleoclimate phases, although the majority of sediments were assigned into the stochastic process. In contrast to bacteria, archaeal communities were consistently assigned to the phylogenetic clustering type in the NRI-NTI model and homogeneous selection (βNTI < −2) in the βNTI model (Supplementary Figure S7), suggesting that the environmental conditions present in JPC1 lead to a convergent composition of archaeal communities with low phylogenetic turnover under consistent selective pressure.

**Figure 7:**
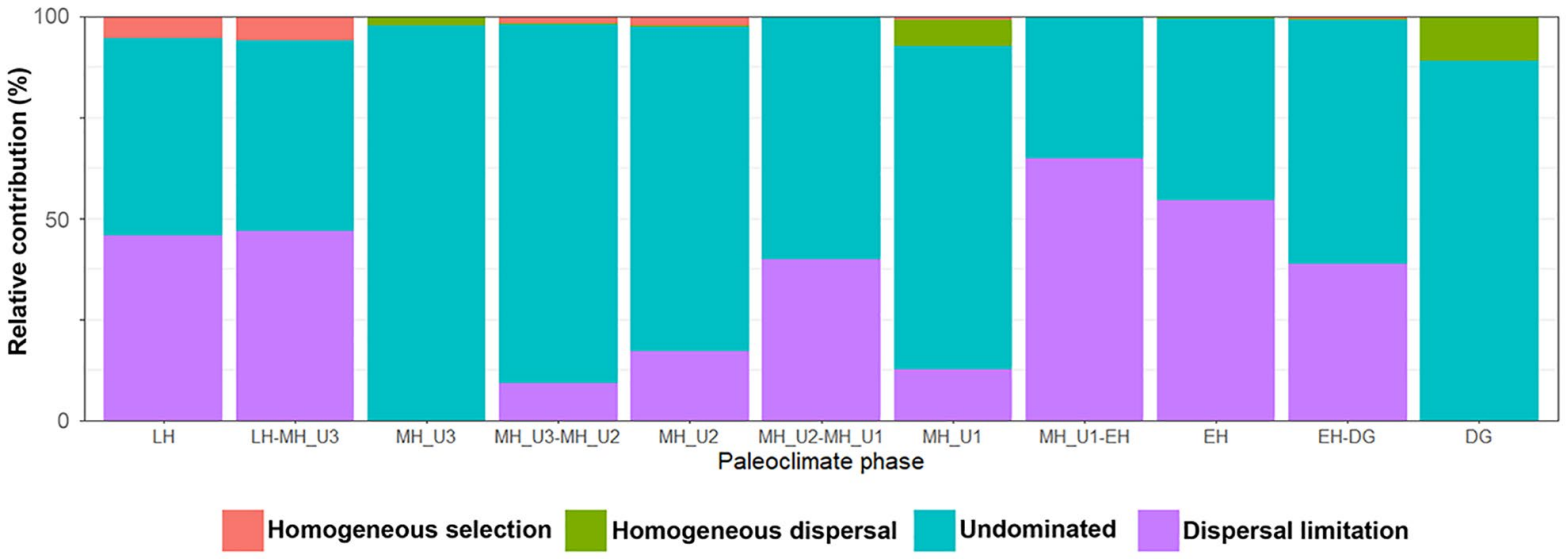
Contribution of homogenizing selection, homogenizing dispersal, undominated, and dispersal limitation processes of bacterial assemblages in different paleoclimate phases.

To identify the factors governing the stochastic community assembly within the bacterial dataset, we extended the phylogenetic analysis to calculate RC_bray_ (Supplementary Figure S8). Our results assigned 3.97% of all pairwise comparisons to homogenizing selection and none to variable selection, similar to the pattern shown in Figure 6B. Within the stochastic processes, most pairs were explained by dispersal limitation (40.1%) or undominated conditions (55.4%), while only 0.55% were assigned to homogenizing dispersal (Supplementary Figure S8). We evaluated the influence of each assembly process within and between the paleoclimate phases in JPC1 (Supplementary Table S4). Except for undominated process interactions, we found that dispersal limitation was the primary factor best explaining the assembly of bacterial communities in JPC1. Moreover, the relative contributions of assembly processes within and between successive paleoclimate phases from DG to LH showed a periodic distribution with three break points at DG, MH_U1, and MH_U3 (Figure 7). These breakpoints were overwhelmed mainly by the undominated process (DG: 88.9%, MH_U1: 80.2%, and MH_U3: 97.7%), with only minor contributions from homogeneous dispersal (DG: 11.1%, MH_U1: 6.3%, and MH_U3: 2.3%) (Figure 7 and Supplementary Table S4).

## Discussion

### Geological and geochemical history in the western Arctic Ocean

The history of the sea-level changes in the Bering Strait has been previously studied (Elias et al., 1996, 1997; Keigwin et al., 2006). The Bering Strait might have been closed by around 11 ky BP (at the end of the last glacial period (80–11 ky BP)) due to the lowered sealevels (Supplementary Figure S9) (Hu et al., 2012). Consequently, most of the BSI could not have flowed into the western Arctic during the last glacial period. However, due to the lack of high-resolution records, our understanding of the microbial assembly process in response to the BSI signal during the post-glacial period (Holocene) has been limited. In this study, we investigated the geochemical properties and microbial biogeography during the paleoclimate phases analyzed in marine sediments at 5–10 cm depth intervals down to 10.07 mbsf.

**Figure 8:**
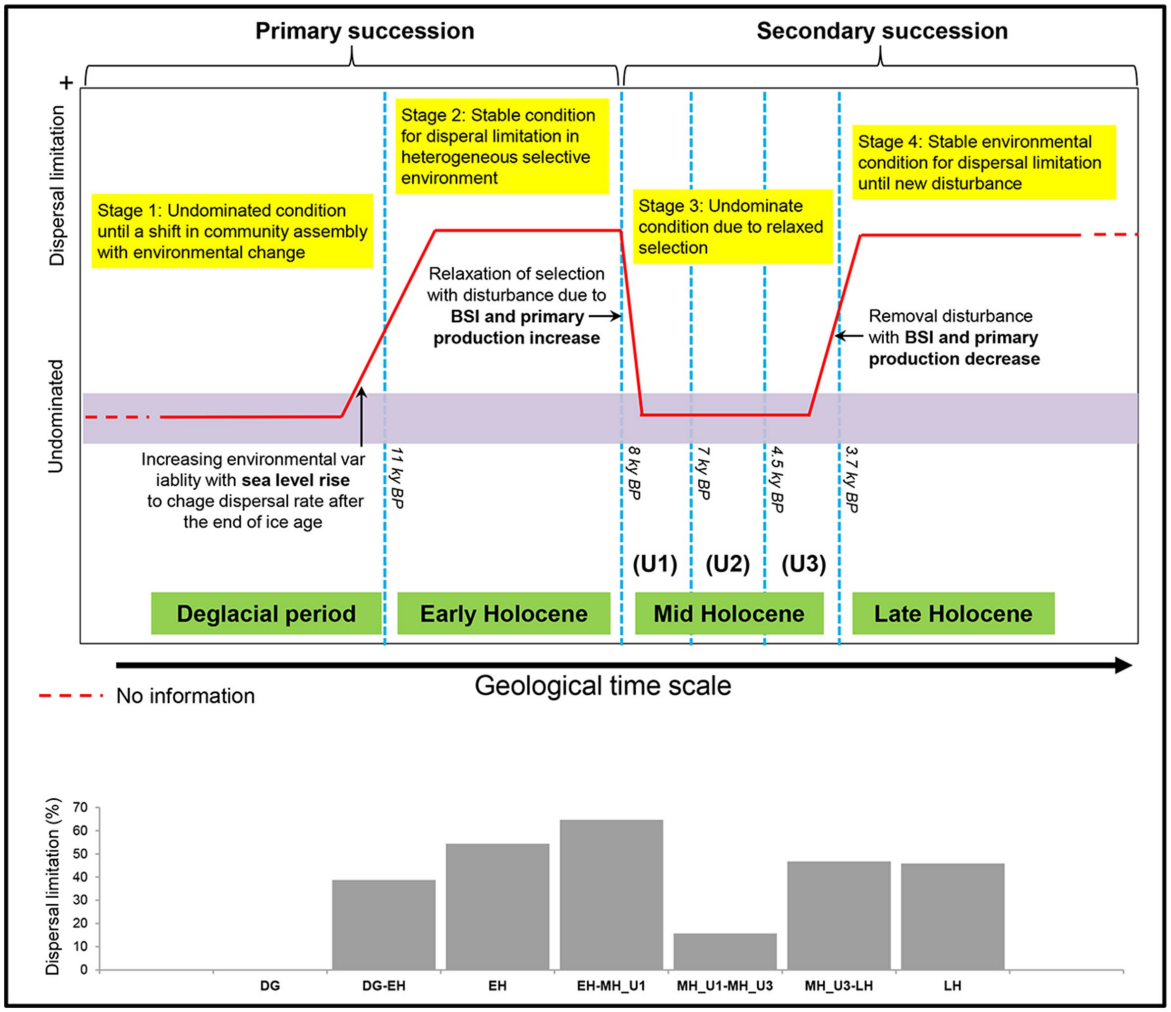
Conceptual model for bacterial community assembly processes at a geological timescale in the Arctic Holocene sediment, reconstructed from the below paleoclimate description of the Chukchi Sea. The used model was modified to scenarios regarding variable selection in Dini-Andreote et al.‘s (2015) successional model. In the ecological assembly perspective, the variable selection and neutral condition are conceptually similar to dispersal limitation (Stegen et al., 2015) and undominate (Dini-Andreote et al., 2015), respectively. Thus, the original terminologies used by Dini-Andreote et al. (2015) were substituted with dispersal limitation and undominated to draw this model.

Pore water profiles of JPC1 (Figure 1A) exhibited a general redox cycling with Mn-Fe reduction and sulfate reduction occurring below oxic surface sediments. On the other hand, the distribution profiles of Ca, Mg, Mn, Fe, and Sr., normalized to Al in the sediment (solid phase) elemental profiles (Figure 1B), indicate changes in the sediment provenance of the western Arctic Ocean, as previously reported (März et al., 2011;Meinhardt et al., 2014; Han et al., 2018). For example, Ca/Al, Mg/Al, and Sr./Al signals are known to be influenced by biogenic carbonate (März et al., 2011), and in JPC1, these showed distribution patterns similar to TOC. The Mn/Al ratio suggests a terrestrial input from Siberian river discharge and coastal erosion in the Chukchi Sea since the opening of the Bering Strait (Meinhardt et al., 2014; Han et al., 2018). Accordingly, the Mn/Al ratio showed an intense signal from EH to MH_U1 in JPC1. The early increase in Mn/Al ratio, and TOC within the EH section in JPC1 may indicate an increased terrestrial input after the Bering Strait opening. In contrast, the decrease of the Mn/Al ratio since the deposition of MH_U1 sediments suggests that TOC mainly originated from pelagic primary production during EH (Figure 1B). The intermediate Mn/Al signal in EH and MH_U1 implies the influence of intensive sea-level rise since the opening of the Bering Strait (Figure 8). The minimized elemental signals, including Mn/Al in the over-consolidated DG sediments, may result from the extent of grounded ice shelves (Lehmann and Jokat, 2021). Marine sediment depositions during intensive sea-level rise may lead to distinguishable sedimentary conditions in further MH_U2, MH_U3, and LH sections (Figure 1). On the contrary, the Fe/Al ratio showed a distribution different from that of Mn/Al, suggesting that Fe may predominantly be bound to oxyhydroxides or terrigenous clay minerals (Meinhardt et al., 2014). Taken together, the pattern of geochemical zones obtained in this study was remarkably consistent with the paleoclimate phases in JPC1, as inferred from Holocene records (e.g., biogenic opal, paleo-temperature, PIP_25_, and sedimentation rate) in the Chukchi Sea (Stein et al., 2017). These findings shed light on the post-glacial changes in the Bering Strait and their potential impact on microbial communities in marine sediments.

Our analysis revealed a high abundance of heterotrophic bacteria, such as Proteobacteria, Atribacteria, and Chloroflexi, in sulfidic sediments (MH_U2 and U3). Notably, these bacterial groups were particularly prominent in the SMTZ sediments within the MH_U2 section, suggesting a competition for organic substrate among heterotrophic bacteria at the boundary layer of SMTZ. Interestingly, we observed a significant presence of cyanobacterial sequences only in the MH_U1, corresponding to the mid-maximum methane (CH_4_) levels (Figure 5). However, it is crucial to note that photosynthetic cyanobacteria are not known as endospore-forming bacteria and cannot remain dormant for thousands of years in subsurface sediments (More et al., 2019). In the present study, we do not argue the case for viable cyanobacteria in the deep biosphere (Puente-Sánchez et al., 2018). Instead, we propose that the detected cyanobacterial DNA signals could be evidence of past algal blooms during the Arctic MH. This assumption aligns with the knowledge that abrupt warming after the last DG period triggered increased productivity and hypoxia in the Gulf of Alaska (Praetorius et al., 2015). The present study supports this idea, given the observed SMTZ signals and the intensity of the BSI since about 8 ky BP (Supplementary Figure S10).

During the MH (3.7–8.0 ky BP), sedimentation rates remarkably increased, coinciding with the deposition of cyanobacteria-enriched sediments around 8.0 ky BP. In general, the redox conditions in marine sediments are governed by oxygen and sulfate levels, which are partly dependent on the sedimentation rate (Orsi, 2018). Slow sedimentation with low organic matter content results in oxic and oligotrophic habitats with deep oxygen penetration. In contrast, fast sedimentation with organic matter-rich sediments leads to rapid oxygen consumption due to the oxidation of organic matter in surface sediments. The deposition of marine sediments in the Chukchi Sea likely began or accelerated after the opening of the Bering Strait around 11 ky BP, with the onset of EH at 7.6 mbsf, above over-consolidated sediments in JPC1. Our study revealed a remarkable increase in geochemical profiles from the EH section (Figure 1), indicating the nutrient-rich Pacific inflow through the Bering Strait into the Chukchi Sea (BSI). However, the relatively lower sedimentation rate in the EH compared to the MH suggests that the BSI might have been still limited to the area of JPC1 during the EH (11–8 ky BP), possibly due to glaciers or low intensity of the BSI. The intensive inflow of the BSI into the Chukchi Sea was inferred from the elevated sedimentation rate and sea-level rise during the MH. In particular, compared to Arctic cold waters, the relatively warmer BSI, might have reduced sea-ice cover between 6.2 and 4.5 ky BP, during which the sedimentation rate reached its maximum (Supplementary Figure S10).

Holocene records from the last 10 ky indicate that abrupt warming coincided with an increased BSI of the nutrient-rich Pacific inflow, possibly leading to enhanced phytoplankton productivity since the MH (from 8 ky BP). The presence of enriched cyanobacterial sequences sharply decreased in sulfate reduction zones starting from the MH_U2 section (since 7 ky BP), suggesting that bacterial degradation of cyanobacterial necromass produced acetate for sulfate-reducing bacteria in Arctic sediments (Müller et al., 2018). Thus, we conclude that high sedimentation with increased biogenic opal within the MH_U1 section represents the growth of cyanobacteria as algal blooms and subsequent hypoxia during 7–8 ky BP. Furthermore, the maximum sedimentation rate until 5.5 ky BP suggests organic matter oxidation in surface sediments under oxygen-limited conditions and the gradual decrease of sedimentation rate since 5.5 ky BP may have affected sulfate-penetration depths during sediment deposition.

### Microbial communities and their assembly mechanism in Arctic Holocene sediments

The fundamental question “do microorganisms have biogeography?” has been answered for many environments. Our research raised the same question in the Arctic subsurface sediments deposited over the Holocene, specifically examining “the relative influence of contemporary environmental factors versus past environmental conditions on present-day distribution patterns” (Martiny et al., 2006). Our findings suggest that microbial biogeography in Arctic Holocene sediments can be explained by the habitat–province theory (Martiny et al., 2006). For example, the diversity and community composition of bacteria and archaea revealed their depth-wise distribution over space (or time) in JPC1 (Supplementary Figures S4, S5). Patterns of NTI-NRI and βNTI models (Figure 6) suggest that the assembly of bacterial communities was influenced by past environmental conditions and subsequent dispersal limitation as described by Martiny et al. (2006) with their hypothesis (multiple provinces–single habitat or past event). Thus, the spatial (or temporal) distribution of microbes in Arctic Holocene sediments, shaped by past oceanographic changes during sediment deposition, confirms that microbes in the subsurface sediments of the Chukchi Sea exhibit microbial biogeography. In particular, archaeal assemblages displayed a relatively simpler composition compared to bacterial assemblages, and the assembly of archaeal communities appears to be less linked to the paleoclimate changes (Supplementary Figure S7) comapred to bacterial communities (Figure 6). On the other hand, More et al. (2021) revealed archaeal communities in subsurface sediments of the northern Red Sea exhibited a strong correlation with paleoenvironmental proxies (not used in our study) such as tetraether index of 86 carbon atoms (TEX_86_) as a proxy for sea surface temperature and the branched over isoprenoid tetraether (BIT) index for terrestrial-derived organic matter. Given microbial habitat preference in diver environments, archaea also can represent the paleoclimate changes in other seafloor conditions.

On a different note, it is crucial to consider that the microbial biogeography in Arctic Holocene sediments may also be influenced by contemporary environmental conditions. For example, biogeochemical traits within the MH SMTZ sediments and microbial dispersal along the environmental gradient during sediment deposition should be viewed differently. As discussed earlier, the effect of variable sedimentation rates during sea-level rise is a driving force for the sulfate-penetrated depth of JPC1, suggesting that our SMTZ signal in the Arctic sediments result from the environmental change during Holocene. This finding is consistent with a previous study in the Black Sea, where the development of SMTZ in subsurface sediments was associated with sea-level rise (Knab et al., 2009). In the Chukchi Sea, microbial motility likely had a minimal contribute much to community composition, except for the oxic surface layer (within the top few centimeters) (Han et al., 2017). Instead, the sedimentation rate seems to be a more influental factor in shaping the deep biosphere (Hinrichs and Inagaki, 2012). Therefore, we emphasize that sedimentation rate may directly or indirectly impact microbial dispersal capacities in Arctic Holocene sediments and the microbial biogeography might reflect the effects of past environmental conditions (paleoclimate change) significantly if dispersal limitation can overwhelm any effect of environmental factors during sediment deposition (Martiny et al., 2006). Our results align with this perspective and demonstrate that dispersal limitation has to be considered the dominant assembly mechanism in the bacterial habitat turnover on a geological timescale. Considering these factors, we can better understand the microbial biogeography in Arctic Holocene sediments and its link to past environmental changes.

### A successional model for bacterial community assembly processes on geological timescale

Our findings regarding the assembly process of the bacterial community under paleoclimate phases were interpreted within an ecological conceptual framework and integrated with a modified conceptual four-stage scenario based on the successional model proposed by Dini-Andreote et al. (2015). Our study supports this conceptual model at a geological timescale (Figure 8). First, bacterial community assembly is not dominated by any process type until the end of the DG section (Stage 1). We consider the DG section as the phase of initial community establishment before a shift in community assembly with changing environmental conditions, although we did not investigate microbial assemblages during the glacial period (uncovered sediments below 10.7 m depth) in the present study. The initial establishment of Stage 1 is disordered during EH (Stage 2) with the increasing contribution in dispersal limitation from the DG period (DG: 0%; EH-DG: 38.7%; EH: 54.4%), which aligns with the end of primary succession in the model established by Dini-Andreote et al. (2015), representing stable condition in heterogeneous selective environment. The high level of dispersal limitation in the EH sharply decreased during the MH (EH-MH_U1: 64.7%; MH_U1: 12.5%; MH_U1-MH_U2: 39.9%; MH_U2: 17.0%; MH_U2-MH_U3: 9.1%; MH_U3: 0%), suggesting that the stable condition of Stage 2 (EH) is linked to a subsequent condition (Stage 3). The model developed by Dini-Andreote et al. (2015) suggests that a disturbance event resulted in the transition from primary (Stage 2) to secondary (Stage 3) succession. In our model, such a disturbance event is thought to play a major role in controlling dispersal limitation. In particular, we assume that the increase in primary production and the BSI since the deposition of the MH_U1 can be considered a disturbance event. The intensity of disturbance (primary production and the BSI) decreases during LH (Stage 4) with a more significant contribution of dispersal limitation (MH_U3-LH: 46.7%; LH: 45.8%). The model suggests that stable environmental conditions might have prevailed during Stage 4 at the end of the primary succession (LH) before a new disturbance (Dini-Andreote et al., 2015). In addition, we observed a relatively high contribution of dispersal limitation in the intermediate pairwise sections, such as between MH_U1 and MH_U2 (MH_U1: 12.5%; MH_U1-MH_U2: 39.9%; MH_U2: 17.0%) (Supplementary Figure S11), which initially seems inconsistent with our conceptual model. However, we postulate that this mismatched signal can be attributed to the variable sedimentation rate, and we believe that the dispersal limitation in the MH_U1–MH_U2 sections may be overestimated due to an abrupt change in sedimentation rate around 7–5.5 ky BP. The mismatched signal disappeared within the maximum sedimentation rate (Supplementary Figure S11), suggesting that the sedimentation rate may shape microbial dispersal capacities and influence the observed microbial biogeography in Arctic Holocene sediments.

Overall, based on the sediment composition and microbial community profiling, we propose that dispersal limitation is an important driving force for the assembly of phylogenetically clustered bacterial communities in Arctic sediments and that BSI influences this process during the Holocene. We neither argue that subseafloor communities are cut off from newly deposited surface sediments (Starnawski et al., 2017) nor explain when (or how) microbial composition is fixed during sedimentation. In addition, *in-situ* microbial interactions during sediment deposition between former (bottom water) and later distributions (underlying surface sediments) were not extensively considered in the present study. This is mainly because oxic or suboxic microbial habitats within the top 10 cm depth of the sediments deposited during the last few hundred years were excluded from JPC1. We focused on the post-burial microbes, presumed to be fixed communities in deeper sediments, to elucidate the microbial biogeography of the past environment in the Arctic deep biosphere. Nevertheless, the comprehensive framework presented in this study significantly improves our understanding of the ecological processes governing community assembly.

The metabarcoding approach has been extensively used in various fields to investigate the dynamics of microbial communities. Although a recent metabarcoding approach using sedimentary ancient DNA, focusing on ancient eukaryotic DNAs, provided data that could potentially reconstruct paleoclimate changes in the Arctic Ocean (De Schepper et al., 2019), its application using prokaryotic DNAs in paleoceanographic studies is still in its infancy. Here, our findings allow us to track the BSI signal using prokaryotic DNA metabarcoding across various sediment depths in the western Arctic Ocean. We demonstrate new perspectives on sedimentary DNAs from microbial prokaryotes regarding climate change.

In conclusion, our study provides valuable insights into the microbial biogeography of Arctic Holocene sediments, highlighting the potential of prokaryotic DNA metabarcoding for studying past environmental changes in the Arctic Ocean. Further research in this direction can enhance our understanding of microbial responses to paleoclimate shifts and their role in shaping marine ecosystems over geological timescales.

## Data availability statement

The datasets presented in this study can be found in online repositories. The names of the repository/repositories and accession number(s) can be found in the article/Supplementary material.

## Author contributions

DH, TR-H, MF, XY, and ME conceived and designed research and contributed sample preparation or analytical tools. DH, J-HK, and J-SR conducted experiments. S-IN led the Arctic expedition and took core sediment. DH, TR-H, J-HK, and J-SR analyzed data. DH, TR-H, J-HK, ME, and KJ wrote the manuscript. All authors contributed to the article and approved the submitted version.

## Funding

This work was supported by the Basic Science Research Program through the National Research Foundation of Korea (NRF) funded by the Ministry of Education (NRF-2018R1D1A3B07041743 and NRF-2022R1F1A1065719). J-HK was supported by the Korea Ministry of Science and ICT (GP2021-009).

## Conflict of interest

The authors declare that the research was conducted in the absence of any commercial or financial relationships that could be construed as a potential conflict of interest.

## Publisher’s note

All claims expressed in this article are solely those of the authors and do not necessarily represent those of their affiliated organizations, or those of the publisher, the editors and the reviewers. Any product that may be evaluated in this article, or claim that may be made by its manufacturer, is not guaranteed or endorsed by the publisher.

## Footnotes

^1^https://www.ncbi.nlm.nih.gov/sra

^2^www.r-project.org
