# Supplementary material for: Influence of sedimentary deposition on the microbial assembly process in Arctic Holocene marine sediments
Source: Front Microbiol. 2023 Aug 28;14:1231839. doi: 10.3389/fmicb.2023.1231839 (PMC10493304; doi:10.3389/fmicb.2023.1231839)
Supplement: Supplementary file 2 [file Data_Sheet_2.docx]

**Influence of sedimentary deposition on the microbial assembly process in Arctic Holocene marine sediments**

**Dukki Han^1*^, Tim Richter-Heitmann^2^, Ji-Hoon Kim^3^, Michael W. Friedrich^2,4^, Xiuran Yin^2,4,5^, Marcus Elvert^4,6^, Jong-Sik Ryu^7^, Kwangchul Jang^8^, and Seung-Il Nam^8^**

^1^Department of Marine Bioscience, Gangneung-Wonju National University, 7, Jukheon-gil, Gangneung-si, Gangwon-do 25457, South Korea

^2^Microbial Ecophysiology Group, Faculty of Biology/Chemistry, University of Bremen, Bremen, Germany

^3^Marine Geology & Energy Division, Korea Institute of Geoscience and Mineral Resources, 124 Gwahak-ro, Yuseong-gu, Daejeon 34132, South Korea

^4^MARUM - Center for Marine Environmental Sciences, University of Bremen, Bremen, Germany

^5^State Key Laboratory of Marine Resource Utilization in South China Sea, Hainan University,

Haikou, China

^6^Organic Geochemistry Group, Faculty of Geosciences, University of Bremen, Bremen, Germany

^7^Department of Earth and Environmental Sciences, Pukyong National University, Busan 48513, South Korea

^8^Division of Glacial Environment Research, Korea Polar Research Institute, Incheon 21990, South Korea

*** Correspondence:** Dukki Han (dukkihan@gwnu.ac.kr)

Keywords: Sedimentary deposition, microbial assembly, Arctic Holocene, marine sediments, eDNA, metabarcoding

**Extra Description**

**S1. Ecological terminology**

**Environmental selection**: Microorganisms are adapted to specific environmental conditions, and different habitats will favor the growth and survival of certain microbial species over others. Environmental factors such as temperature, pH, nutrient availability, and other physical and chemical characteristics play a significant role in shaping microbial communities.

**Ecological drift**: Like all living organisms, microorganisms can be subject to random fluctuations in population size due to chance events. These random changes, known as ecological drift, can lead to variations in microbial community composition over time, particularly in small or isolated populations.

**Dispersal ability**: The ability of microorganisms to move from one location to another is an essential factor in determining their distribution. Some microorganisms may have high dispersal abilities, allowing them to spread over long distances through air, water, or other vectors, while others may be more restricted in their movement.

**Speciation**: Over time, microorganisms can evolve into distinct species through genetic mutations and natural selection. Speciation can lead to the development of unique microbial communities in different locations, especially if populations become isolated from one another.

| **Terminology** | **Description** | **Reference** |
| --- | --- | --- |
| Deterministic process | Assembly process of community composition is influenced by abiotic factor (environmental selection) and species interaction (antagonistic and synergistic). Deterministic process can be categorized into homogeneous (low compositional turnover) or variable selections (high compositional turnover). | (Stegen, Lin, Fredrickson, & Konopka, 2015) |
| Stochastic process | Assembly process of community composition is under unpredictable disturbance in species movement (ecological drift or dispersal) or population size. Stochastic process can be separated into dispersal limitation or homogenous dispersal. | (Stegen et al., 2015) |
| The net relatedness index (NRI) | “A standardized measure of the mean pairwise phylogenetic distance of taxa in a sample, relative to a phylogeny of an appropriate species pool, and quantifies overall clustering of taxa on a tree” | (Webb, Ackerly, McPeek, & Donoghue, 2002) |
| The nearest taxon index (NTI) | “A standardized measure of the phylogenetic distance to the nearest taxon for each taxon in the sample and quantifies the extent of terminal clustering, independent of deep level clustering” | (Webb et al., 2002) |
| Beta Nearest Taxon Index (βNTI) | A measure of the phylogenetic beta diversity between samples (the between-assemblage analogs of NTI) | (Webb et al., 2002) |
| Phylogenetic turnover model | Homogeneous selection in deterministic process: significantly less than expected phylogenetic turnover (βNTI < -2); variable selection in deterministic process: significantly more than expected phylogenetic turnover (βNTI > +2); stochastic process: the lack of deviation (-2 < βNTI < +2) | (Dini-Andreote, Stegen, van Elsas, & Salles, 2015; Stegen, Lin, Konopka, & Fredrickson, 2012) |

**S2. Core description**

JPC1 exhibits three distinct lithological units, which comprise bioturbated mud with dark mottles (0-760 cm), laminated mud (760-910.5 cm) and massive mud (910.5-1028.5 cm). These main sediment structures in the lithological units of JPC1 have probably been formed during the sedimentation (e.g., lamination) and/or early deposition process (e.g., bioturbation). Additionally, we also observed a vertical crack between 606 and 676.5 cm and contorted lamination in the depth between 783 and 835 cm. These minor features observed within the bioturbated and laminated units appear to form during post-depositional processes. Given that the vertical crack and the contorted lamination occur solely in the geochemical zone IV affected by methanogenesis, both features might be interpreted as the sedimentary structures deformed by methane production and subsequent gas migration.

During the Expedition, physical properties (black line: wet bulk density, WBD, red line: volumetric specific magnetic susceptibility, MS) of JPC1 were determined on whole cores at 1 cm intervals using a multi-sensor-core-logger (MSCL-S, Geotek Ltd.). These include volume-specific magnetic susceptibility (Bartington MS-2 loop sensor), wet bulk density (gamma ray attenuation), and p-wave velocity (500 kHz plate transducers).


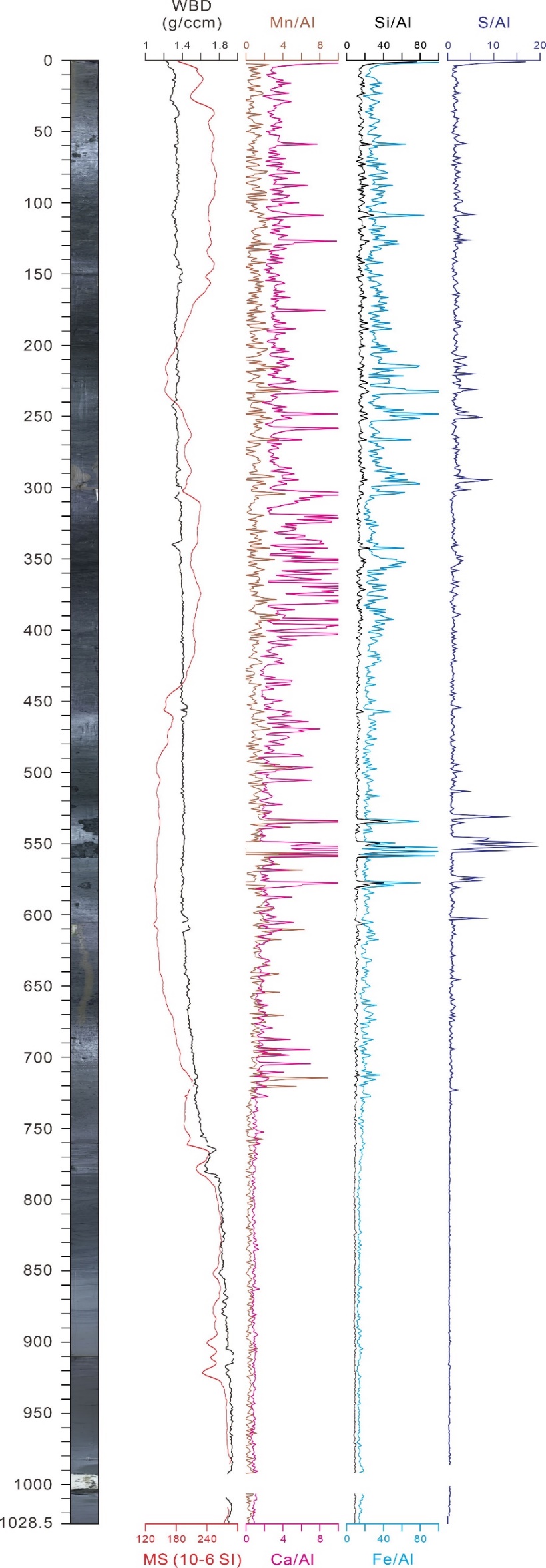


Depth (cmbsf)

**S3. Geochemical experiment (Han et al., 2017)**

Sulfate (SO_4_) concentrations were measured at depths of 0.2, 0.7, 1.2, 1.5, 2.3, 2.8, 3.3, 3.8, 4.3, 4.8, 5.3, 5.8, 6.3, 6.8, and 7.8 mbsf, and methane (CH_4_) concentrations were measured at depths of 1.5, 3.0, 4.6, 6.1, 7.6, 9.1, 10.4 mbsf. Pore fluid was extracted by Rhizone technique and was collected with 24 ml acid-prewashed syringes. Subsamples for the anion analysis were collected directly from the syringes into acid-prewashed Nalgene high-density polyethylene bottles after the extracted pore fluid was filtered through a 0.2 µm disposable polytetrafluoroethylene filter. Sulfate was analyzed by ion chromatography (ICS-1500, Dionex) with an AS-40 auto sampler at the Korea Institute of Geoscience and Mineral Resources (KIGAM). Reproducibility was better than 3%, as estimated by repeated measurements of standard analysis. For headspace gas analyses, a 3 ml sediment sample was taken with a cut-off 5 ml plastic syringe from the freshly exposed end of each core section and extruded into a 20 ml glass serum vial. Following the methods described before (Choi et al., 2013; Kim et al., 2012), 2 ml of saturated NaCl was added to each vial, which was then sealed with a 10 mm thick septum and a metal crimp cap. Headspace gas was extracted post cruise by heating the sediment samples at 60°C for 30 min at KIGAM, following the procedure described before (Pimmel & Claypool, 2001). HS gases were injected into an Agilent Technologies 7890A gas chromatograph with flame ionization detector (FID) at KIGAM to analyze hydrocarbon composition. Reproducibility from repeated standard analysis was better than 5%.

**S4. Metabarcoding and qPCR**

**1) Primer list**

| **Primer set for** | **Primer sequences** | **Annealing temp.** | **Reference** |
| --- | --- | --- | --- |
| Archeal 16S rRNA qPCR | Arc_967F: 5´ AATTGGCGGGGGAGCAC 3´  Arc_1060R: 5´ GGCCATGCACCWCCTCTC 3´ | 60℃ | (Cadillo‐Quiroz et al., 2006) |
| Bacterial 16S rRNA qPCR | Bac338F: 5´ ACTCCTACGGGAGGCAGC 3´  Bac518R: 5´ ATTACCGCGGCTGCTGG 3´ | 60℃ | (Lane, 1991) |
| Archeal 16S rRNA sequencing | Forward: 5´ **TCGTCGGCAGCGTCAGATGTGTATAAGAGACAG(Illumina’s adapter region)** CCGACGGTGAGRGRYGAA(Archeal 16S rRNA sequencing region) 3´  Reverse: 5´**GTCTCGTGGGCTCGGAGATGTGTATAAGAGACAG(Illumina’s adapter region)** TTMGGGGCATRCIKACCT(Archeal 16S rRNA sequencing region) 3´ | 55℃ | (Baker, Smith, & Cowan, 2003) |
| Bacterial 16S rRNA sequencing | Forward: 5´ **TCGTCGGCAGCGTCAGATGTGTATAAGAGACAG(Illumina’s adapter region)** CCTACGGGNGGCWGCAG(Bacterial 16S rRNA sequencing region) 3´  Reverse: 5´ **GTCTCGTGGGCTCGGAGATGTGTATAAGAGACAG(Illumina’s adapter region)** GACTACHVGGGTATCTAATCC(Bacterial 16S rRNA sequencing region) 3´ | 55℃ | (Herlemann et al., 2011) |

**2) PCR conditions**

**For metabarcoding:** Illumina MiSeq platform (Macrogen, Seoul, South Korea) was used to sequence archaeal and bacterial 16S rRNA genes of eDNAs through metabarcoding-based sequencing with two-step PCR (amplicon and index PCR) according to Illumina’s instruction. The first amplicon PCRs were carried out for eDNAs in triplicates using KAPA HiFi Hotstart ReadyMix PCR kit (KAPA BioSystems, MA, USA) with archeal and bacterial 16S rRNA sequencing primer sets. The 1^st^ PCR amplicons were purified using the Qiaquick PCR purification kit (Qiagen, CA, USA), and then the next index PCRs proceeded using the PCR amplicons under Illumina’s protocol. Concentrations of the index PCRs were measured by Qubit 2.0 Fluorometer (Invitrogen, CA, USA) after amplicon purification. The purified amplicons were all mixed in equimolar amounts to construct Illumina MiSeq library and subjected to sequencing.

**For qPCRs:** Abundance of archeal and bacterial 16S rRNA genes were quantified using SYBR Premix Ex Taq™ with a Thermal Cycler Dice Real Time System (Takara Bio Inc., Shiga, Japan) according to the manufacturer’s specifications. Information of used primer sequences and their specific annealing temperatures for the qPCR was previously described (above primer list). The quantification standard for archeal and bacterial 16S rRNA genes consisted of ten-fold serial dilution of a known amount of gDNA in *Methanobacterium congolense* (anaerobic archaea, DSM7095) and *Escherichia coli* (DSM30083), respectively. qPCRs for the 16S rRNA genes in samples and standards were performed in triplicates with the following conditions: initial denaturation at 94°C for 3 min, followed by 40 cycles of denaturation at 94°C for 20 s, annealing at 60°C for 30 s, and elongation at 72°C for 30 s. At the end of each run, a dissociation melt curve of the PCR product was determined to verify amplicon specificity.

**For Calculation of 16S rRNA gene copy number:** The number of 16S rRNA genes was calculated from *E. coli*  and *M. congolense* genomic DNA. We used the followed formula: 16S rRNA genes per nanogram gDNA=[6.022 × 10^23^ (avogadro’s constant) /(base pairs of genome × 660 (average mass of 1 bp of dsDNA) × 10^9^ (conversion factor)] × number of 16S rRNA genes per genome. The genome sizes used were 5,038,133 bp for *E. coli* (Meier-Kolthoff et al., 2014) and 2,451,457 bp for *M. congolense* (Tejerizo et al., 2017). The numbers of 16S rRNA genes per genome used for *E. coli* and *M. congolense* were seven and three, respectively. These values were collected from the ribosomal RNA operon copy number database (https://rrndb.umms.med.umich.edu/) (Stoddard, Smith, Hein, Roller, & Schmidt, 2015).

**S5. Phylogenetic analysis**

**1) Phylogenetic index calculation**

We used Phylocom (Webb, Ackerly, & Kembel, 2008) to calculate NRI, NTI, and βNTI (nonpairwise) values according to User’s manual (Ver. 4.1). Briefly, Phylocom requires a Newick-format phylogeny and a sample data as input files. We prepared the phylogeny file using Mothur (Schloss et al., 2009) according to the MiSeq SOP (Kozich, Westcott, Baxter, Highlander, & Schloss, 2013). The sample data consists of 3 columns in plain text file following sample name, species abundance, and species name (same as in the phylogeny’s species name). For operating Phylocom calculation, we selected ‘Phylogeny shuffle’ as a null model, which randomizes phylogenetic relationships among species and randomized 999 runs. We further calculated the RC_bray_ with the previous description (Stegen et al., 2013) and new βNTI (pairwise) using ‘picante’ package (Kembel et al., 2010) in R ([www.r-project.org](http://www.r-project.org)).

**2) NRI-NTI model (Amaral-Zettler et al., 2011; Webb et al., 2002)**

We estimate an influence between biotic and abiotic forces on community assembly with two phylogenetic structuring patterns using NRI-NTI model.

| **Phylogenetic patterns** | **Description** |
| --- | --- |
| Over-dispersion (NRI and NTI < 0) | The assembly of communities under biological interaction (biotic factors including symbiosis–competition or immigration–emigration) |
| Clustering (NRI and NTI > 0) | In contrast to over-dispersion, the assembly process influenced by abiotic factors (habitat specificity or environmental selection) |

**3) βNTI- RC_bray_ model (Feng et al., 2018; Stegen et al., 2015)**

Bray-Curtis-based Raup–Crick metric (RCbray) and βNTI were calculated together to differentiate ecological scenarios in the comprehensive framework.

- Variable selection (βNTI > 2) leads *“divergent community composition with high phylogenetic turnover due to variable selective pressure in environmental condition.”*

- Homogeneous selection (βNTI < -2) leads *“convergent community composition with low phylogenetic turnover under a consistent selective pressure in environmental condition”*

- Homogeneous dispersal (｜βNTI｜ < 2, RCbray < -0.95) leads *“compositional similarity due to high dispersal rate and low phylogenetic turnover.”*

- Dispersal limitation (｜βNTI｜ < 2, RCbray > 0.95) leads *“dissimilar community composition under low dispersal rate and high phylogenetic turnover. Dispersal limitation is conceptually similar with variable selection.”*

- Undominated (｜βNTI｜ < 2, ｜RCbray｜ < 0.95) is referred to *“no single dominant dispersal and selection in community assembly.”*


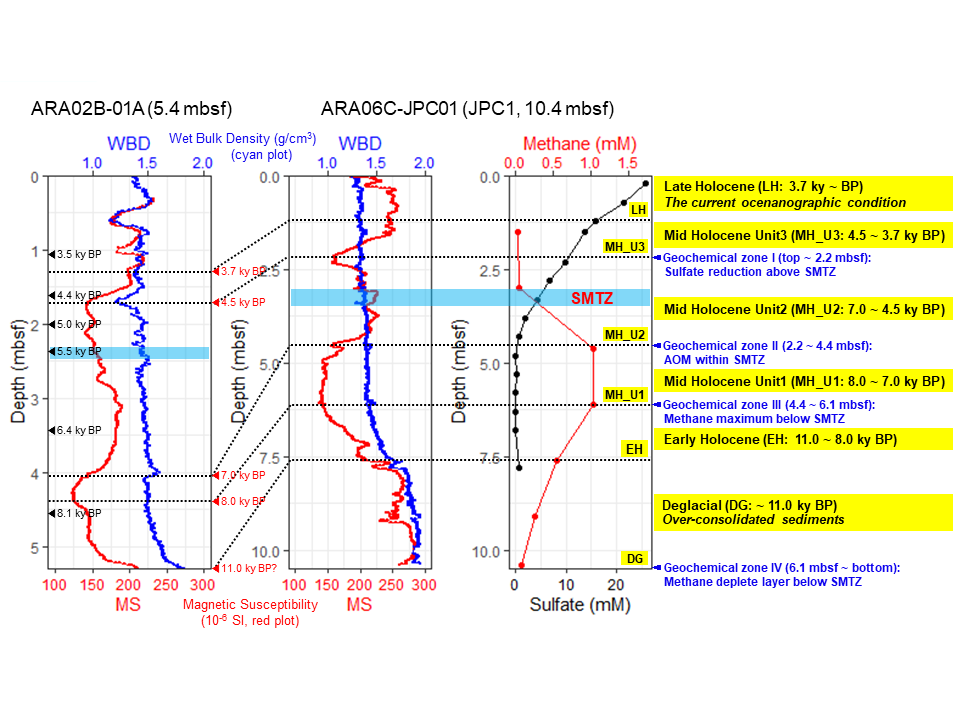
**Supplementary Figures and Tables**

Supplementary Figure S1. Correlation between JPC1 and ARA2B-1A (Stein et al., 2017) based on profiles of magnetic susceptibility and wet bulk density and concentrations of sulfate and methane in the core of JPC1. Description of JPC1 under paleoclimate phase was referred from Stein *et al.* (2017).


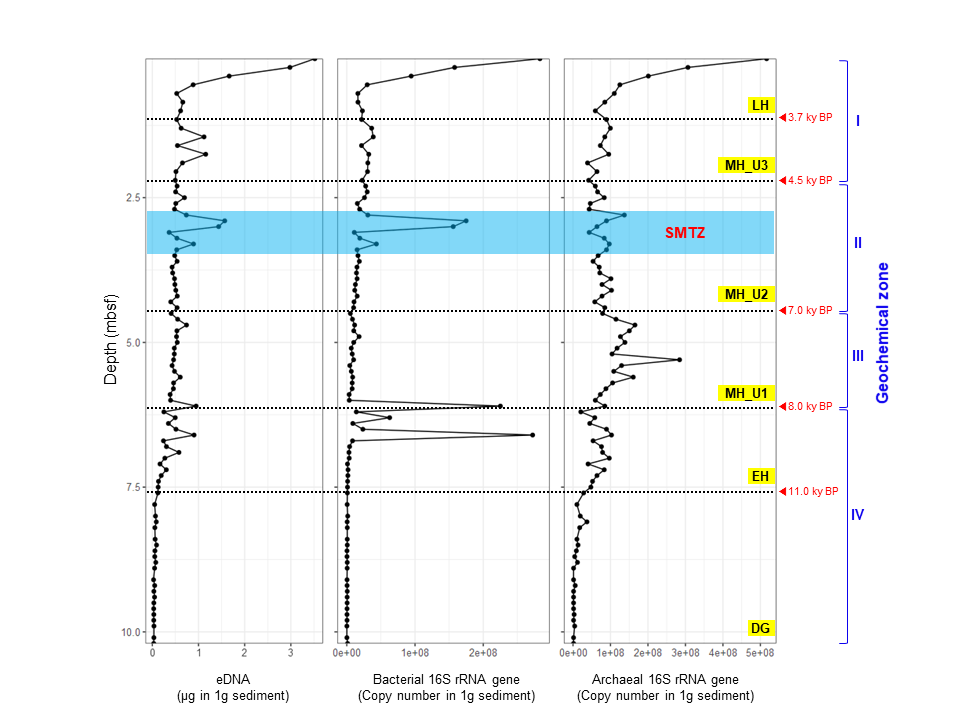


Supplementary Figure S2. Profiles of eDNA, Bacterial 16S rRNA gene, and Archaeal 16S rRNA gene in JPC1.


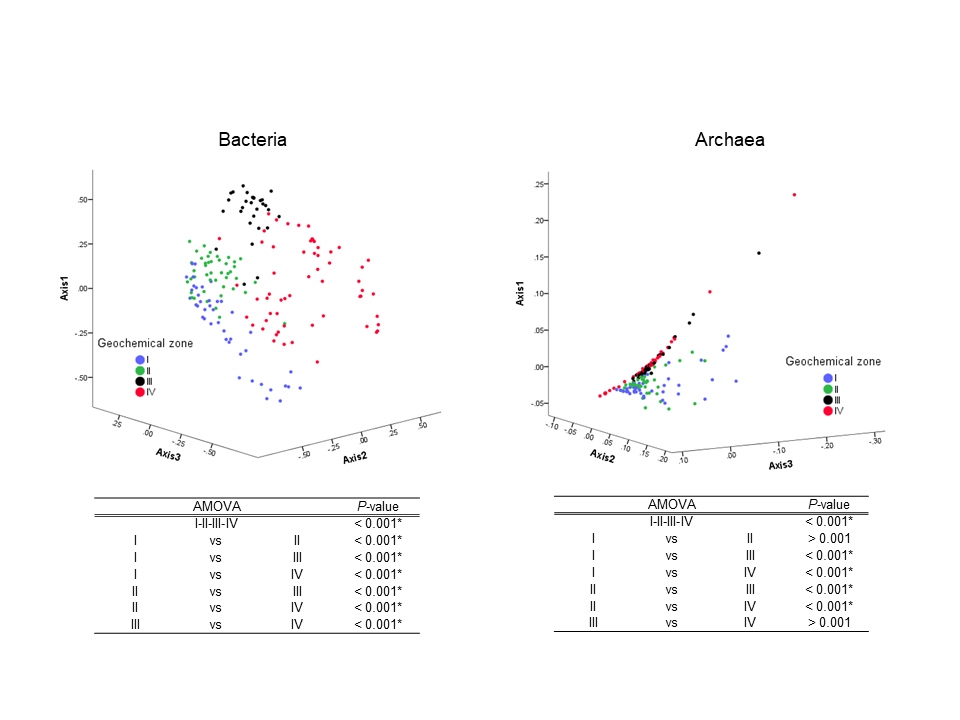


Supplementary Figure S3. Beta diversity of microbial communities in different geochemical zones in JPC1.


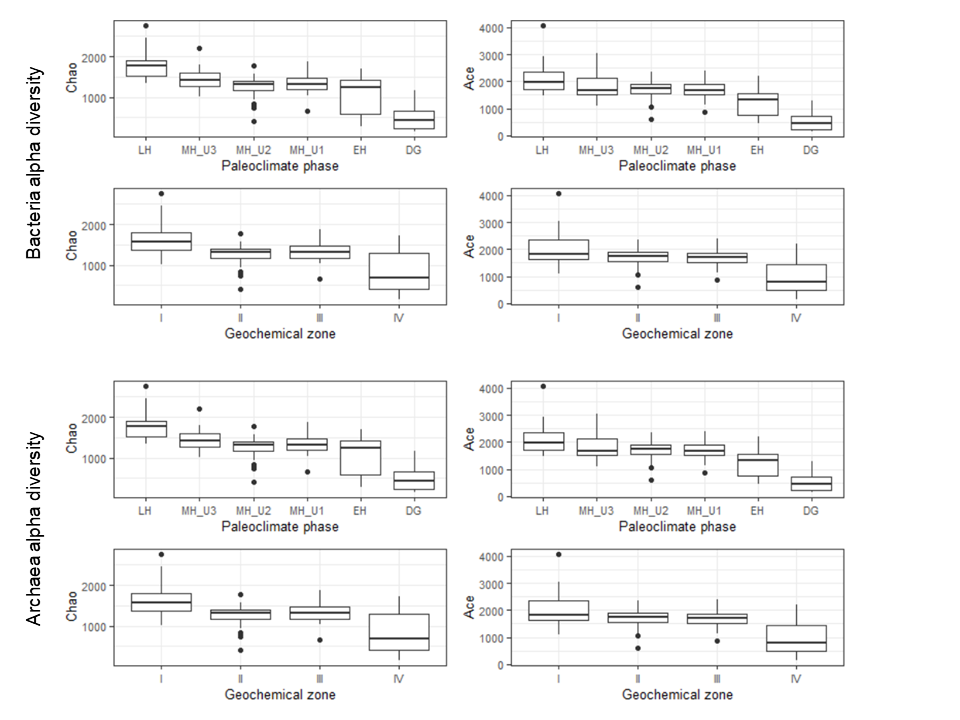


Supplementary Figure S4. Alpha diversity of microbial communities in different paleoclimate phases in JPC1.


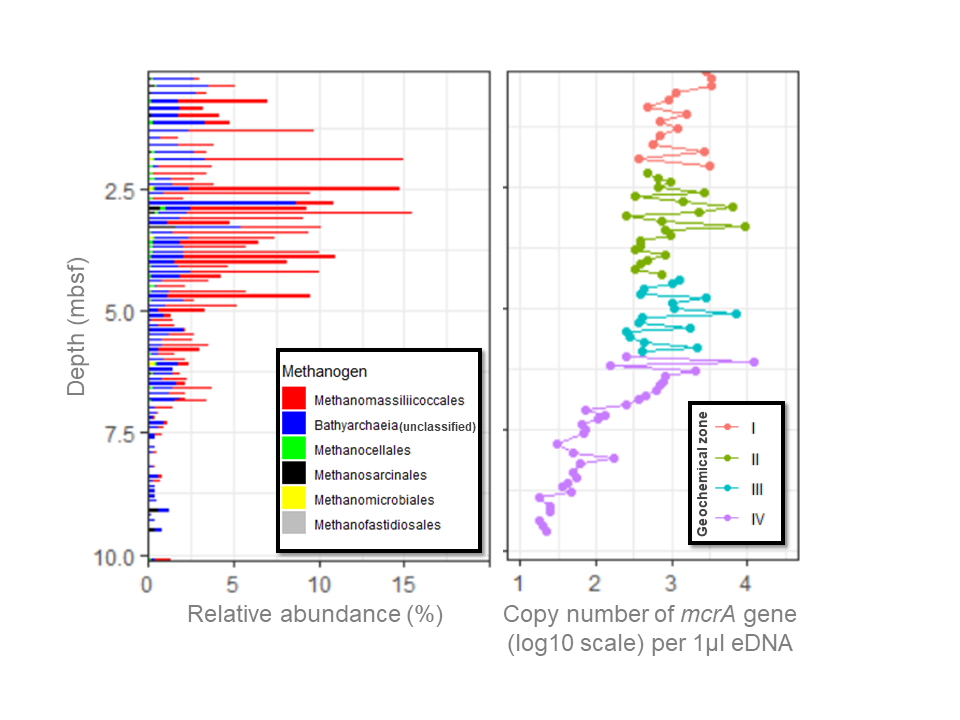


Supplementary Figure S5. Distribution of methanogenic archaea and *mcrA* gene in JPC1.


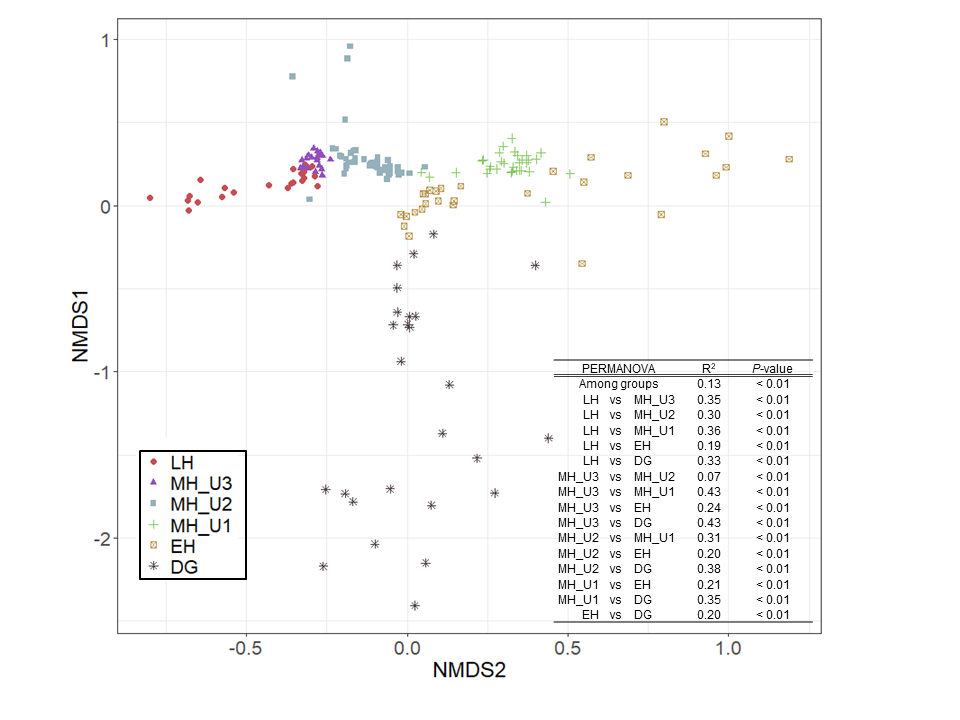


Supplementary Figure S6. Paleoclimate-specific clustering displayed by top 300 bacterial OTUs. Beta diversity was visualized using NMDS. The significance of differences in bacterial OTUs between each pair of paleoclimate phases was determined by PERMANOVA (*P* < 0.001).


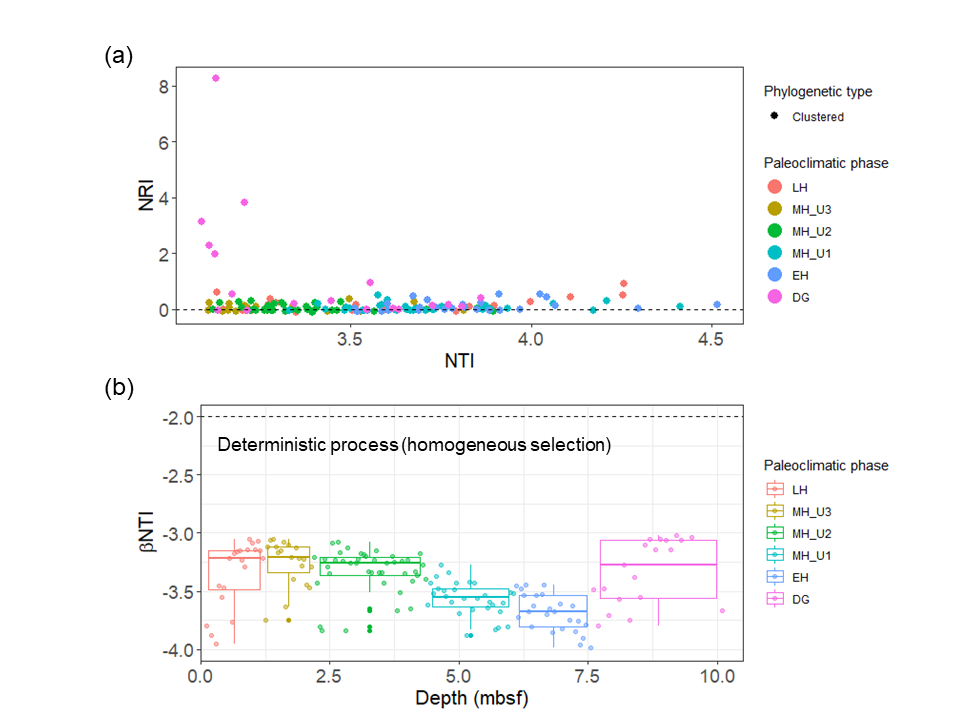


Supplementary Figure S7. Ecological assembly perspectives for archaeal communities in JPC1. (a) Identification of the phylogenetic type using NTI and NRI indexes and (b) into stochastic and deterministic assembly processes using βNTI index under paleoclimate phase.


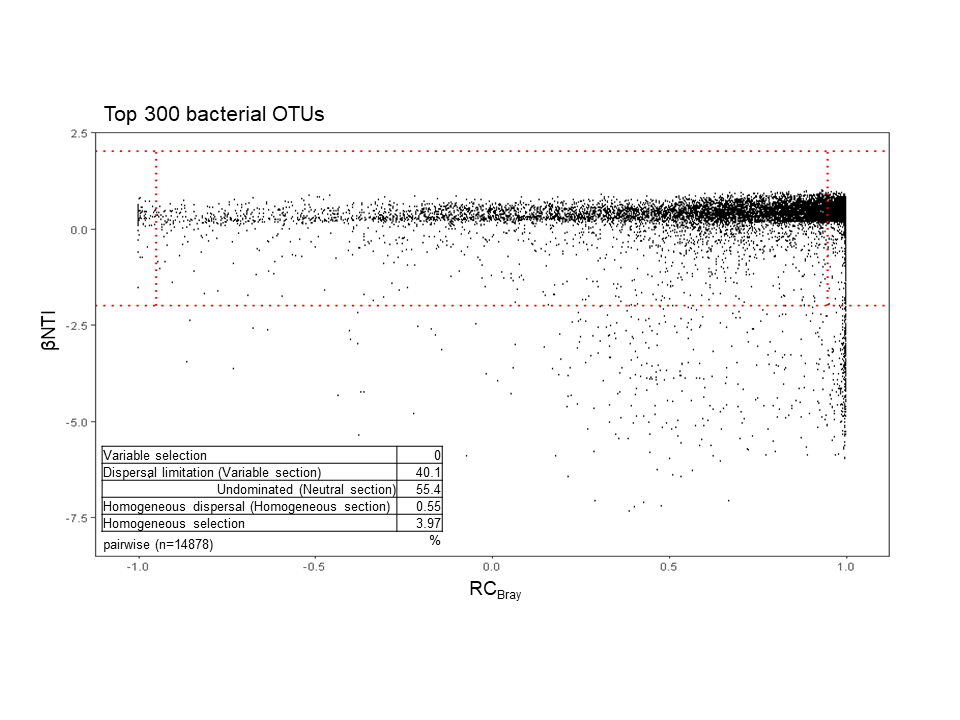


Supplementary Figure S8. The five categories of assembly processes in the comprehensive ecological model (Stegen et al., 2015) using the top 300 bacterial OTUs.


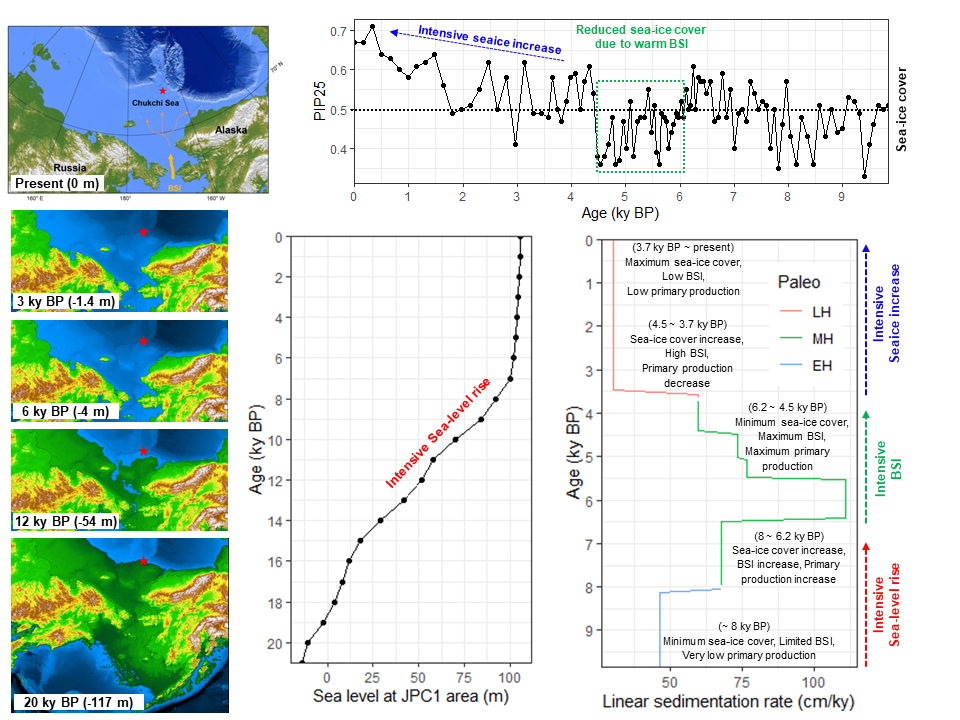


Supplementary Figure S9. Paleoclimate description of the Chukchi Sea. Sea-level record was referred from the land bridge animation (Manley, 2002) and re-calculated with the current sea-level of 106 m at the coring location of JPC1 (red colored star on the map). Sea-ice coverage (PIP_25_) and linear sedimentation rate were from Stein *et al.* (2017).


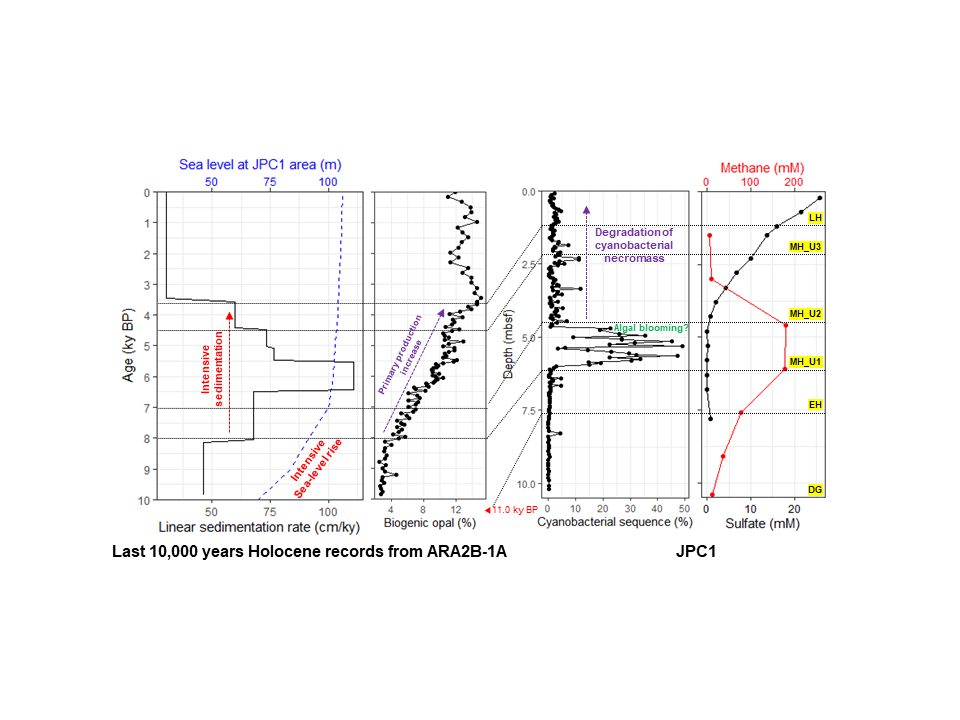


Supplementary Figure S10. Comparison between the relative abundance of cyanobacteria in JPC1 and last 10 ky BP Holocene records from the Chukchi Sea (Stein et al., 2017).


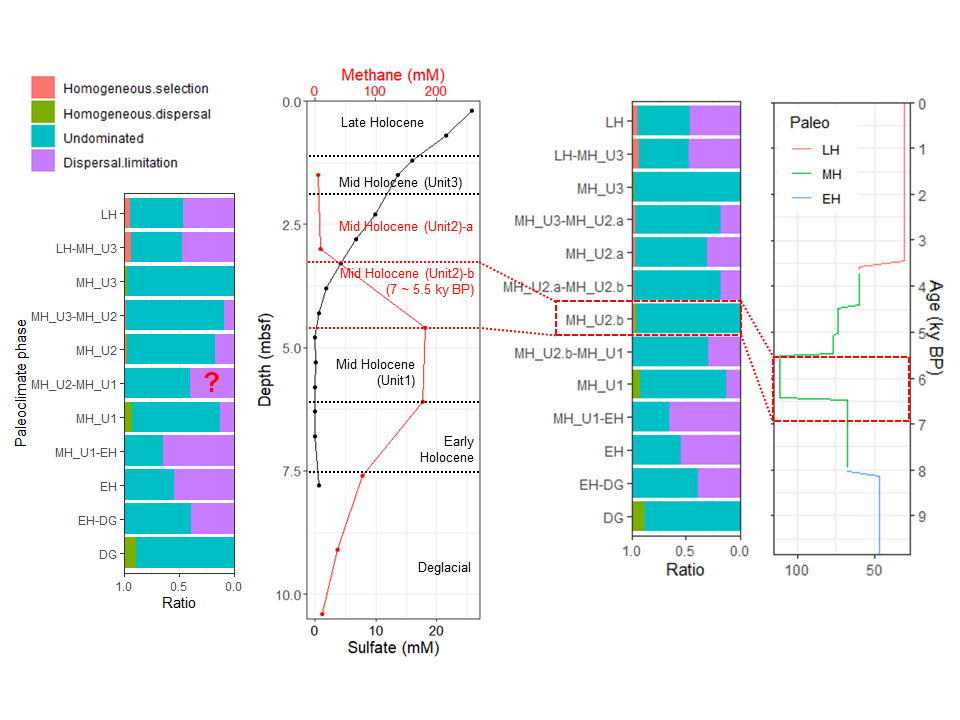


Supplementary Figure S11. Contribution of homogenizing selection, homogenizing dispersal, undominated, and dispersal limitation processes of bacterial assemblages under the modified paleoclimate phase.

**Supplementary Tables**

Supplementary Table S1. The statistical separations among geochemical or paleoclimate groups. The significant differences among groups were determined by MRPP.

| MRPP | Among groups | Geochemical zones (*P* < 0.01) | | | | Paleoclimate phases (*P* < 0.01) | | | | | |
| --- | --- | --- | --- | --- | --- | --- | --- | --- | --- | --- | --- |
|  | Within group | I | II | III | IV | LH | MH_U3 | MH_U2 | MH_U1 | EH | DG |
|  | Mean distance (δ) | 0.05 | 0.08 | 0.07 | 0.23 | 0.04 | 0.04 | 0.08 | 0.07 | 0.37 | 0.05 |
|  | Number of samples | 7 | 8 | 5 | 15 | 4 | 3 | 8 | 5 | 5 | 10 |

Supplementary Table S2. The statistical separations among bacterial and archaeal communities in paleoclimate phases, supported by AMOVA. Significant differences were marked with an asterisk (*P* < 0.001).

| Paleoclimate phases | | |  | AMOVA (*P*-value) | |
| --- | --- | --- | --- | --- | --- |
|  |  |  |  | Bacteria | Archaea |
| LH-MH_U3-MH_U2-MH_U1-EH-DG | | |  | < 0.001* | < 0.001* |
| LH | vs | MH_U3 |  | < 0.001* | > 0.001 |
| LH | vs | MH_U2 |  | < 0.001* | > 0.001 |
| LH | vs | MH_U1 |  | < 0.001* | < 0.001* |
| LH | vs | EH |  | < 0.001* | < 0.001* |
| LH | vs | DG |  | < 0.001* | < 0.001* |
| MH_U3 | vs | MH_U2 |  | < 0.001* | > 0.001 |
| MH_U3 | vs | MH_U1 |  | < 0.001* | < 0.001* |
| MH_U3 | vs | EH |  | < 0.001* | < 0.001* |
| MH_U3 | vs | DG |  | < 0.001* | < 0.001* |
| MH_U2 | vs | MH_U1 |  | < 0.001* | < 0.001* |
| MH_U2 | vs | EH |  | < 0.001* | < 0.001* |
| MH_U2 | vs | DG |  | < 0.001* | < 0.001* |
| MH_U1 | vs | EH |  | < 0.001* | > 0.001 |
| MH_U1 | vs | DG |  | < 0.001* | < 0.001* |
| EH | vs | DG |  | < 0.001* | < 0.001* |

Supplementary Table S3. The variation of phylogenetic patterns in paleoclimate phases.

| Paleoclimate  phase | Phylogenetic type (%) | | |
| --- | --- | --- | --- |
|  | Over-dispersed | Clustered | Ambiguous |
| LH | 74 | 17 | 9 |
| MH_U3 | 100 | 0 | 0 |
| MH_U2 | 98 | 0 | 2 |
| MH_U1 | 3 | 97 | 0 |
| EH | 36 | 46 | 18 |
| DG | 0 | 100 | 0 |

Supplementary Table S4. Mapping of ecological assembly processes of the bacterial community within or between pairs of different paleoclimate phases.

| **Paleoclimate phase** | | | **Homogeneous selection (%)** | **Homogeneous dispersal (%)** | **Undominated (%)** | **Dispersal limitation (%)** |
| --- | --- | --- | --- | --- | --- | --- |
| Within (n=2,597) | | | **1.6** | **3.0** | **74.4** | **21.7** |
| LH | | (n=253) | 5.5 | 0.0 | 48.6 | 45.8 |
| MH_U3 | | (n=171) | 0.0 | 2.3 | 97.7 | 0.0 |
| MH_U2 | | (n=946) | 2.2 | 0.4 | 80.3 | 17.0 |
| MH_U1 | | (n=496) | 1.0 | 6.3 | 80.2 | 12.5 |
| EH | | (n=406) | 0.0 | 0.7 | 44.8 | 54.4 |
| DG | | (n=325) | 0.0 | 11.1 | 88.9 | 0.0 |
| Between (n=12,281) | | | **4.5** | **0.0** | **51.5** | **44.0** |
| LH | MH_U3 | (n=437) | 5.9 | 0.0 | 47.4 | 46.7 |
| LH | MH_U2 | (n=1012) | 2.3 | 0.0 | 41.3 | 56.4 |
| LH | MH_U1 | (n=736) | 0.0 | 0.0 | 32.9 | 67.1 |
| LH | EH | (n=667) | 1.3 | 0.0 | 22.8 | 75.9 |
| LH | DG | (n=598) | 12.4 | 0.0 | 61.4 | 26.3 |
| MH_U3 | MH_U2 | (n=836) | 1.7 | 0.2 | 89.0 | 9.1 |
| MH_U3 | MH_U1 | (n=608) | 0.0 | 0.0 | 55.4 | 44.6 |
| MH_U3 | EH | (n=551) | 0.5 | 0.0 | 37.4 | 62.1 |
| MH_U3 | DG | (n=494) | 26.3 | 0.0 | 52.4 | 21.3 |
| MH_U2 | MH_U1 | (n=1408) | 0.1 | 0.0 | 59.9 | 39.9 |
| MH_U2 | EH | (n=1276) | 0.5 | 0.0 | 37.9 | 61.6 |
| MH_U2 | DG | (n=1144) | 21.9 | 0.0 | 57.2 | 20.9 |
| MH_U1 | EH | (n=928) | 0.1 | 0.1 | 35.1 | 64.7 |
| MH_U1 | DG | (n=832) | 0.7 | 0.0 | 76.0 | 23.3 |
| EH | DG | (n=754) | 0.7 | 0.1 | 60.5 | 38.7 |

**References**

Amaral-Zettler, L. A., Zettler, E. R., Theroux, S. M., Palacios, C., Aguilera, A., & Amils, R. (2011). Microbial community structure across the tree of life in the extreme Rio Tinto. *The ISME journal, 5*(1), 42.

Baker, G., Smith, J. J., & Cowan, D. A. (2003). Review and re-analysis of domain-specific 16S primers. *Journal of microbiological methods, 55*(3), 541-555.

Cadillo‐Quiroz, H., Bräuer, S., Yashiro, E., Sun, C., Yavitt, J., & Zinder, S. (2006). Vertical profiles of methanogenesis and methanogens in two contrasting acidic peatlands in central New York State, USA. *Environmental microbiology, 8*(8), 1428-1440.

Choi, J., Kim, J.-H., Torres, M. E., Hong, W.-L., Lee, J.-W., Yi, B. Y., . . . Lee, K. E. (2013). Gas origin and migration in the Ulleung Basin, East Sea: Results from the Second Ulleung Basin Gas Hydrate Drilling Expedition (UBGH2). *Marine and Petroleum Geology, 47*, 113-124.

Dini-Andreote, F., Stegen, J. C., van Elsas, J. D., & Salles, J. F. (2015). Disentangling mechanisms that mediate the balance between stochastic and deterministic processes in microbial succession. *Proceedings of the National Academy of Sciences of the United States of America, 112*(11), E1326-E1332.

Feng, Y., Chen, R., Stegen, J. C., Guo, Z., Zhang, J., Li, Z., & Lin, X. (2018). Two key features influencing community assembly processes at regional scale: Initial state and degree of change in environmental conditions. *Molecular ecology, 27*(24), 5238-5251.

Han, D., Nam, S.-I., Kim, J.-H., Stein, R., Niessen, F., Joe, Y. J., . . . Hur, H.-G. (2017). Inference on Paleoclimate Change Using Microbial Habitat Preference in Arctic Holocene Sediments. *Scientific reports, 7*(1), 9652.

Herlemann, D. P., Labrenz, M., Jürgens, K., Bertilsson, S., Waniek, J. J., & Andersson, A. F. (2011). Transitions in bacterial communities along the 2000 km salinity gradient of the Baltic Sea. *The ISME journal, 5*(10), 1571-1579.

Kembel, S. W., Cowan, P. D., Helmus, M. R., Cornwell, W. K., Morlon, H., Ackerly, D. D., . . . Webb, C. O. (2010). Picante: R tools for integrating phylogenies and ecology. *Bioinformatics, 26*(11), 1463-1464.

Kim, J.-H., Torres, M. E., Choi, J., Bahk, J.-J., Park, M.-H., & Hong, W.-L. (2012). Inferences on gas transport based on molecular and isotopic signatures of gases at acoustic chimneys and background sites in the Ulleung Basin. *Organic Geochemistry, 43*, 26-38.

Kozich, J. J., Westcott, S. L., Baxter, N. T., Highlander, S. K., & Schloss, P. D. (2013). Development of a dual-index sequencing strategy and curation pipeline for analyzing amplicon sequence data on the MiSeq Illumina sequencing platform. *Applied and Environmental Microbiology, 79*(17), 5112-5120.

Lane, D. (1991). 16S/23S rRNA sequencing In: Stackebrandt E, Goodfellow M, editors. Nucleic acid techniques in bacterial systematics. In: New York: John Wiley and Sons.

Manley, W. F. (2002). Postglacial flooding of the Bering Land Bridge: A geospatial animation. *INSTAAR, University of Colorado, Boulder*.

Meier-Kolthoff, J. P., Hahnke, R. L., Petersen, J., Scheuner, C., Michael, V., Fiebig, A., . . . Goodwin, L. A. (2014). Complete genome sequence of DSM 30083T, the type strain (U5/41T) of Escherichia coli, and a proposal for delineating subspecies in microbial taxonomy. *Standards in genomic sciences, 9*(1), 1-19.

Pimmel, A., & Claypool, G. (2001). Introduction to shipboard organic geochemistry on the JOIDES Resolution.

Schloss, P. D., Westcott, S. L., Ryabin, T., Hall, J. R., Hartmann, M., Hollister, E. B., . . . Robinson, C. J. (2009). Introducing mothur: open-source, platform-independent, community-supported software for describing and comparing microbial communities. *Applied and Environmental Microbiology, 75*(23), 7537-7541.

Stegen, J. C., Lin, X., Fredrickson, J. K., Chen, X., Kennedy, D. W., Murray, C. J., . . . Konopka, A. (2013). Quantifying community assembly processes and identifying features that impose them. *The ISME journal, 7*(11), 2069-2079.

Stegen, J. C., Lin, X., Fredrickson, J. K., & Konopka, A. E. (2015). Estimating and mapping ecological processes influencing microbial community assembly. *Frontiers in microbiology, 6*, 370.

Stegen, J. C., Lin, X., Konopka, A. E., & Fredrickson, J. K. (2012). Stochastic and deterministic assembly processes in subsurface microbial communities. *The ISME journal, 6*(9), 1653-1664.

Stein, R., Fahl, K., Schade, I., Manerung, A., Wassmuth, S., Niessen, F., & Nam, S. I. (2017). Holocene variability in sea ice cover, primary production, and Pacific‐Water inflow and climate change in the Chukchi and East Siberian Seas (Arctic Ocean). *Journal of Quaternary Science, 32*(3), 362-379.

Stoddard, S. F., Smith, B. J., Hein, R., Roller, B. R., & Schmidt, T. M. (2015). rrn DB: improved tools for interpreting rRNA gene abundance in bacteria and archaea and a new foundation for future development. *Nucleic acids research, 43*(D1), D593-D598.

Tejerizo, G. T., Kim, Y. S., Maus, I., Wibberg, D., Winkler, A., Off, S., . . . Schlüter, A. (2017). Genome sequence of Methanobacterium congolense strain Buetzberg, a hydrogenotrophic, methanogenic archaeon, isolated from a mesophilic industrial-scale biogas plant utilizing bio-waste. *Journal of biotechnology, 247*, 1-5.

Webb, C. O., Ackerly, D. D., & Kembel, S. W. (2008). Phylocom: software for the analysis of phylogenetic community structure and trait evolution. *Bioinformatics, 24*(18).

Webb, C. O., Ackerly, D. D., McPeek, M. A., & Donoghue, M. J. (2002). Phylogenies and community ecology. *Annual review of ecology and systematics, 33*(1), 475-505.
